# Electroacupuncture alleviates functional dyspepsia by modulating the vagus nerve to regulate duodenal microbiota and suppress TWEAK/Fn14/NF-κB and arachidonic acid metabolic pathways

**DOI:** 10.3389/fimmu.2026.1746351

**Published:** 2026-03-09

**Authors:** Xueping Zhang, Xinxin Hu, Jiaxuan Li, Xiaojing Song, Chengxiang Wang, Yang Chen, Suowei Wu, Lixin Ma, Wenqi Jiang, Ran Cai, Xiaolan Su, Wei Wei

**Affiliations:** 1 Department of Gastroenterology, Beijing Key Laboratory of Functional Gastrointestinal Disorders Diagnosis and Treatment of Traditional Chinese Medicine, Wangjing Hospital, China Academy of Chinese Medical Sciences, Beijing, China; 2 Graduate School, Hubei University of Chinese Medicine, Wuhan, Hubei, China; 3 Institute of Acupuncture and Moxibustion, China Academy of Chinese Medical Sciences, Beijing, China; 4 Graduate School, Beijing University of Chinese Medicine, Beijing, China; 5 School of Medical Technology, Beijing Institute of Technology, Beijing, China

**Keywords:** arachidonic acid metabolism, electroacupuncture, functional dyspepsia, multi-omics, TWEAK/Fn14/NF-κB pathway

## Abstract

**Aim:**

This study investigates the therapeutic mechanisms of electroacupuncture (EA) in regulating the vagal nerve for functional dyspepsia (FD) using an integrated multi-omics approach.

**Methods and results:**

A rat model of FD was established via iodoacetamide gavage combined with tail-clamp stress. Rats were randomly assigned to five groups (n=6 per group): control (CON), model (MOD), electroacupuncture (EA), subdiaphragmatic vagotomy and electroacupuncture (SDV+EA), and subdiaphragmatic vagotomy (SDV). EA was administered at ST36 (Zusanli) and ST37 (Shangjuxu) for 20 minutes per session, once daily for 14 days. EA treatment restored vagal tone, improved sympathovagal balance, and enhanced gastrointestinal motility in FD model rats. 16S rDNA sequencing revealed that EA modulated vagus nerve-dependent changes in the relative abundance of 12 microbial taxa, including *f_Lactobacillaceae* and *f_Peptostreptococcaceae*. Crucially, the vagotomy procedure significantly attenuated EA’s restorative effects on these microbial populations. Metabolomics identified 24 differential metabolites regulated by EA through the vagus nerve, including Cholesta-3,5-dien-7-one, Licofelone, Digoxigenin, 7-Hydroxymethotrexate, Hydroxymethylbilane, among others. Similarly, subdiaphragmatic vagotomy largely reversed the normalizing effects of EA on these metabolite levels. Transcriptomics, on the other hand, identified 23 differential genes, including Prss22, Lypd3, and Tnfrsf12a. KEGG analysis of differential metabolites and differential genes suggested that arachidonic acid metabolism may represent a potential therapeutic target for EA in the treatment of FD through vagus nerve modulation. Mechanistic analyses of the key differentially expressed gene *Tnfrsf12a* and the arachidonic acid metabolic pathway demonstrated that EA attenuated inflammatory responses by suppressing TWEAK/Fn14/NF-κB pathway activation and arachidonic acid metabolism, leading to decreased levels of TNF-α, IL-1β, IL-6, and PGE_2_. Importantly, the anti-inflammatory effects of EA were significantly attenuated in the SDV+EA group, confirming that vagal integrity is essential for EA to fully exert its suppressive action on these key inflammatory pathways and mediators.

**Conclusion:**

EA ameliorates FD by modulating vagal nerve activity, concurrently suppressing TWEAK/Fn14/NF-κB pathway activation and arachidonic acid metabolism, thus attenuating duodenal low-grade inflammation in FD model rats. These findings demonstrate the potential of EA as an effective therapeutic intervention for FD.

## 1 Introduction

Functional dyspepsia (FD) is a non-organic disorder characterized by gastroduodenal dysfunction and is one of the most prevalent disorders of gut-brain interaction (DGBI), affecting approximately 8-12% of the global population (1). While the pathogenesis of FD remains incompletely understood, current evidence suggests a multifactorial nature involving gastrointestinal dysmotility, visceral hypersensitivity, dysregulated mucosal immune responses, and alterations in gut microbiota (2). In recent years, a growing body of research has recognized the duodenum as a key site in the pathogenesis of FD. FD patients often exhibit low-grade inflammation in the duodenal mucosa, characterized by increased infiltration of eosinophils and mast cells, upregulated expression of inflammatory cytokines, and impaired mucosal barrier function. These alterations can activate sensory nerve endings, heighten visceral sensitivity, disrupt gastrointestinal motility regulation, and contribute to intestinal microbial dysbiosis, thereby inducing or exacerbating FD symptoms (3–6).

Under the contemporary biopsychosocial medical model, accumulating evidence has highlighted the pivotal role of brain-gut axis signaling in FD pathogenesis. The vagus nerve (VN), serving as the primary bidirectional communication pathway between the central nervous system and the gastrointestinal tract, plays a crucial role in regulating gastrointestinal motility and secretory functions (7). Clinical studies have demonstrated reduced vagal tone in FD patients, contributing to impaired gastric accommodation and motility dysfunction (8–10). Reduced vagal function may weaken the vagus-mediated cholinergic anti-inflammatory pathway, which in turn could contribute to the persistence of duodenal mucosal inflammation (11). Vagal nerve stimulation (VNS) has been shown to enhance vagal activity, promote gastric peristalsis, reduce inflammation, and ameliorate clinical symptoms in FD patients (2, 12). Preclinical studies further support these findings, demonstrating that VNS restores vagal tone, normalizes autonomic nervous system activity, modulates gastric slow-wave frequency, and downregulates pro-inflammatory cytokine expression in FD animal models (13–15). These collective findings strongly support VN modulation as a promising therapeutic strategy for FD management, suggesting that its therapeutic effects may be mediated through the regulation of duodenal inflammatory responses.

Electroacupuncture (EA) is a therapeutic modality that combines traditional acupuncture with modern electrical stimulation. This involves the application of controlled electrical currents through filiform needles inserted at specific acupoints to enhance treatment efficacy. EA is recognized for its favorable safety profile, clinical effectiveness, and minimal adverse effects. Previous studies have shown that EA exerts anti-inflammatory effects through activation of the vagal-adrenal axis (16). Specifically, EA modulates adrenergic signaling pathways, suppresses norepinephrine secretion, enhances vagal activity, and restores sympathovagal balance in FD models, thereby ameliorating immune-inflammatory responses and attenuating visceral hypersensitivity (15, 17–19). However, the specific molecular mechanisms by which EA alleviates duodenal inflammation through vagal nerve modulation remain to be further elucidated.

In this study, we employed an integrative multi-omics approach combining 16S rDNA sequencing, untargeted metabolomics, and transcriptomics to elucidate the mechanisms by which EA alleviates duodenal low-grade inflammation in FD through VN modulation, with subsequent experimental validation of key differential genes and metabolic pathways. The integration of gut microbiota profiling, transcriptomics, and metabolomics enables comprehensive identification of phenotype-associated microbial taxa, differentially expressed genes, and differential metabolites, thereby elucidating core regulatory metabolic pathways and molecular networks (20). This approach further clarifies the mechanisms underlying EA’s attenuation of duodenal low-grade inflammation in FD and the pivotal regulatory role of the VN in these processes, providing a scientific foundation for understanding EA’s therapeutic effects on FD pathophysiology.

## 2 Materials and methods

### 2.1 Animals

A total of 32 male Sprague Dawley (SD) rats (5 days old) were purchased from Sibeifu (Beijing) Biotechnology Co., Ltd. and housed in standard cages at the Institute of Basic Theory for Chinese Medicine, China Academy of Chinese Medical Sciences. Before weaning (postnatal day 21), pups were maintained with their dams. After weaning at 3 weeks of age, the rats were group-housed (6 animals per cage) under controlled conditions. All animals were maintained under standardized specific pathogen-free (SPF) conditions with controlled environmental parameters: temperature (20-24°C), relative humidity (40–50%), and a 12-hour light/dark cycle (lights on at 08:00). Throughout the experimental period, the rats had ad libitum access to conventional rodent lab chow and water. All experimental protocols were reviewed and approved by the Ethics Committee of the Experimental Animal Center of the Institute of Basic Theory for Chinese Medicine, China Academy of Chinese Medical Sciences (Approval No.: IBTCMCAS21-2404-02).

### 2.2 FD model establishment and experimental grouping

After a 5-day acclimation period, 32 rats (initially 5 days old) were randomly divided, with 6 rats assigned to the control group (CON). Beginning on postnatal day 10, the remaining 26 rats received daily oral gavage with 0.2 mL of 0.1% iodoacetamide (IA) in 2% sucrose for 6 consecutive days, while the CON group received 0.2 mL of 2% sucrose. Following the induction period, all rats were maintained under standard housing conditions until 7 weeks of age. At week 7, FD rats were subjected to tail-clamping stimulation using surgical forceps to clamp the distal one-third of their tails, leading to defensive and fighting behaviors. Each clamping session lasted for 30 minutes, four times a day, for 7 days. The modeling process was completed by 8 weeks of age (13, 21, 22). All animals survived the modeling procedure. Two rats from the FD group were randomly selected for HE staining of the gastric antrum and duodenal tissues to verify the success of the modeling. After successful modeling, the remaining 24 FD rats were randomly divided into four groups: model group (MOD), electroacupuncture group (EA), subdiaphragmatic vagotomy and electroacupuncture group (SDV+EA), and subdiaphragmatic vagotomy group (SDV), with each group consisting of 6 animals.

### 2.3 Surgical procedures

At week 8, rats in the SDV+EA and SDV groups underwent subdiaphragmatic vagotomy. Following an overnight fast, the rats were anesthetized using a small animal anesthesia system (R550, RWD Life Science, China) with 3% isoflurane. After removing the fur from the abdomen, the area was disinfected, and a midline incision was made to expose the stomach and the lower surface of the diaphragm. The bilateral VN were identified under the diaphragm and severed (23). The rats were allowed a one-week recovery period.

### 2.4 Intervention

One week post-surgery, rats received daily EA treatment under 3% isoflurane anesthesia. Following sterile skin preparation with 75% ethanol, sterile acupuncture needles (0.3 mm × 25 mm; Zhongyan Taihe) were inserted bilaterally to a depth of 5 mm at ST36 (Zusanli) and ST37 (Shangjuxu) (24). Needles were connected to an electronic stimulator (Huatuo SDZ-IIB) delivering dense-disperse waves (2/100 Hz alternating) at an intensity eliciting hind-limb tremor (25). Each session lasted 20 minutes, administered every morning (09:00-11:00) for 14 consecutive days.

**Figure 1:**
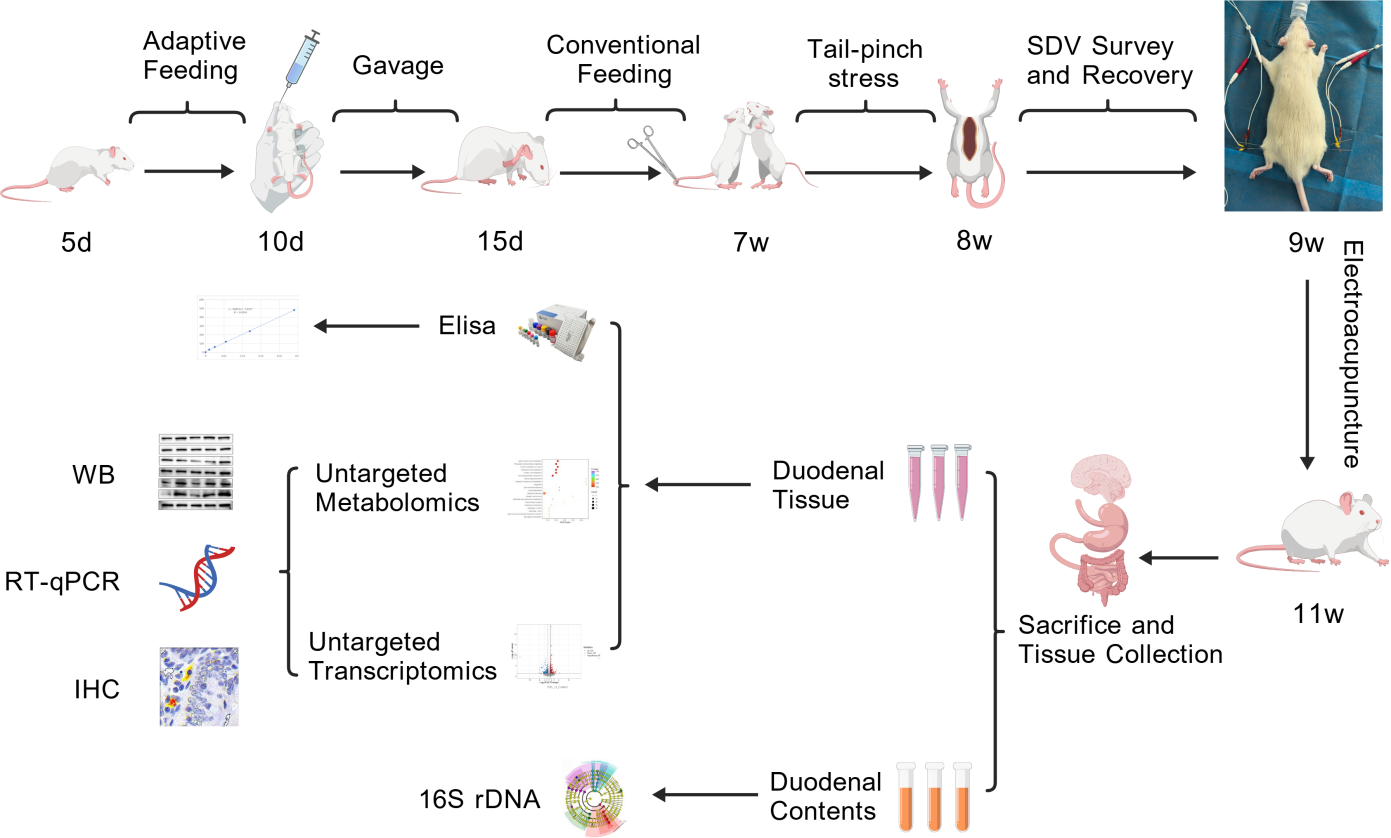
Experimental study design. This picture was created with BioGDP.com.

- CON: Rats received no experimental interventions but were provided with sufficient feed and clean drinking water, with regular bedding replacement and cage cleaning.
- MOD, SDV: Animals were housed under identical conditions as the CON group. During the EA and SDV+EA treatments, these groups were anesthetized for 20 minutes in the anesthesia system.
- EA, SDV+EA: Animals were housed under identical conditions as the CON group. Bilateral EA was performed at ST36 and ST37 using sterile needles inserted vertically to a 5 mm depth. The experimental design schematic is presented in **Figure 1** (26).

### 2.5 Sample collection

A semi-solid paste was prepared by dissolving 10 g of carboxymethyl cellulose sodium, 16 g of skim milk powder, 8 g of corn starch, 8 g of sucrose, and 2 g of activated charcoal in 250 mL of distilled water (27). After the EA intervention, rats in each group were fasted (with free access to water) for 12 hours and orally administered the semi-solid paste (3ml per rat) (28). Rats were anesthetized 50 minutes after gavage, followed by cervical dislocation. Laparotomy was subsequently performed to harvest the stomach and small intestine. The stomach was opened along the greater curvature to expose the mucosa, gently rinsed with ice-cold saline to remove luminal contents, and blotted dry on filter paper. The gastric antrum and proximal duodenum were dissected: one portion was immediately fixed in 4% paraformaldehyde for histology, and the remainder was snap-frozen in liquid nitrogen and stored at -80 °C for subsequent analyses.

### 2.6 Outcome measures

#### 2.6.1 General condition assessment

All animals were assessed for mental and autonomous activities, fur condition, and growth status. Body weight and 24-hour food intake were measured pre-intervention (week 9) and post-treatment (week 11).

#### 2.6.2 Heart rate variability analysis

Post-intervention, rats were anesthetized and positioned supine for standard limb lead II ECG recording via subcutaneous needle electrodes (right forelimb, right hindlimb, left hindlimb). Continuous 5-minute recordings were acquired using a physiological data acquisition system. HRV analysis is based on the principle that the beat-to-beat fluctuations in R-R intervals reflect the dynamic modulation of autonomic nervous system activity on the sinoatrial node, which regulates heart rate. To minimize noise and ensure accuracy, stable frequency bands were selected. Spectral analysis was conducted using the LabChart 8.0 HRV module to quantify key frequency-domain and time-domain parameters: High-frequency (HF) primarily reflects parasympathetic (vagal) activity; the low-frequency to high-frequency ratio (LF/HF) is considered an indicator of sympathovagal balance; and the root mean square of successive differences (RMSSD), a time-domain measure, is a robust marker of short-term vagal-mediated heart rate regulation. Together, these indices provide a non-invasive assessment of autonomic function relevant to the intervention.

#### 2.6.3 Gastric emptying rate and intestinal propulsion rate

Following administration of the charcoal-labeled nutrient paste and subsequent laparotomy, the stomach and intestine were excised for quantitative analysis. The total stomach weight was recorded, the black semi-solid paste was cleaned out, and the empty stomach weight was recorded. The gastric emptying rate (%) = [1- (total stomach weight - empty stomach weight)/semi-solid paste weight] × 100%. The evaluation of intestinal propulsion rate was conducted as follows: a segment of the intestine from the pylorus to the ileocecal junction was excised and laid flat on a smooth surface without stretching. The length from the pylorus to the furthest point of carbon powder propulsion was measured and the total length of the intestinal segment were recorded. The intestine propulsion rate (%) = farthest distance of carbon powder/total length of intestine × 100% (29).

#### 2.6.4 ELISA

Duodenal levels of tumor necrosis factor-α (TNF-α), interleukin-1β (IL-1β), IL-6, and prostaglandin E_2_ (PGE_2_) were quantified using ELISA kits (TNF-α: JM-01587R1; IL-1β: JM-01454R1; IL-6: JM-01597R1; PGE_2_: JM-01475R1, Jingmei Biotech) according to manufacturer protocols, with optical density measurements acquired via microplate reader at 450 nm.

#### 2.6.5 HE staining of gastric antrum and duodenum

Paraffin-embedded gastric antrum and duodenal tissues were sectioned at 4-5 μm thickness, and baked at 60 °C for 30 min to ensure firm adhesion. For hematoxylin and eosin staining, sections were immersed in hematoxylin solution for 5–8 min, and sections were then counterstained with eosin for 1–3 min. After staining, sections were dehydrated and cleared, and then mounted with neutral resin. Histological alterations were evaluated by light microscopy.

#### 2.6.6 16S rDNA sequencing

Total genomic DNA was extracted from samples using the VAMNE Stool/Soil DNA Extraction Kit-BOX2 (Vazyme). The integrity and purity of the extracted DNA were quantitatively assessed. Qualified DNA samples were subjected to first-round PCR amplification with target-region-specific primers and amplification enzyme. After purification of the first-round PCR products using magnetic beads, a second round of PCR amplification was performed. The resulting amplicons were purified again with magnetic beads, followed by concentration quantification using a Qubit fluorometer and target fragment size verification by agarose gel electrophoresis. Sequencing libraries were constructed using the Illumina TruSeq^®^ DNA PCR-Free Sample Preparation Kit. Qualified libraries were sequenced on the Illumina NovaSeq 6000 platform with paired-end reads. Sequences were clustered into operational taxonomic units (OTUs) at a 97% similarity threshold, and subsequent alpha and beta diversity analyses were conducted. Microbial taxa with differential abundances were identified using linear discriminant analysis (LDA) effect size (LEfSe).

#### 2.6.7 Untargeted metabolomic profiling of duodenal contents

Frozen samples were ground and extracted with pre-chilled 70% aqueous methanol at 1,500 r/min for 5 min, followed by incubation on ice for 15 min. The mixture was centrifuged at 12,000 r/min for 10 min at 4 °C. A 300 μL aliquot of the supernatant was transferred to a fresh tube, incubated at –20 °C for 30 min, and centrifuged again at 12,000 r/min for 3 min at 4 °C. The supernatant was carefully transferred into a sample vial insert for LC-MS analysis. Metabolite separation was performed on a Waters ACQUITY Premier HSS T3 column using an ultra-performance liquid chromatography-mass spectrometry system. Raw mass spectrometry data were converted to mzXML format using ProteoWizard, followed by peak detection, alignment, and retention time correction with XCMS. Multivariate statistical analyses including principal component analysis (PCA) and partial least squares discriminant analysis (PLS-DA) and orthogonal partial least squares discriminant analysis (OPLS-DA) were performed, and metabolic pathways were annotated using the KEGG database. Differential metabolites were identified based on a variable importance in projection (VIP) score > 1 and *P* < 0.05.

#### 2.6.8 Transcriptomic profiling of duodenal tissue

Frozen tissue samples were homogenized in 1 mL VeZol Reagent, and chloroform was added for phase separation. After centrifugation at 12,000 ×g for 15 min at 4 °C, the upper aqueous phase was collected. Total RNA was isolated using a magnetic bead–based purification method, and RNA concentration and integrity were assessed prior to library preparation. mRNA was enriched from total RNA using Oligo(dT) magnetic beads based on the poly(A) tail structure of eukaryotic mRNA. The enriched mRNA was fragmented in fragmentation buffer and used as template for first-strand cDNA synthesis with random hexamer primers. Second-strand cDNA was synthesized using dNTPs as substrates, followed by adapter ligation. The ligation products were purified and size-selected using magnetic beads, and then amplified by PCR to generate the final cDNA library. Library quality was assessed by initial quantification using a Qubit fluorometer and fragment size analysis with a Qsep400 high-throughput biofragment analyzer. Only libraries with the expected fragment size proceeded to sequencing. Qualified libraries were sequenced on an Illumina platform to generate 150 bp paired-end reads. Differentially expressed genes were identified using thresholds of |log_2_Fold Change| ≥ 1 and *P* < 0.05.

#### 2.6.9 Reverse transcription-quantitative polymerase chain reaction (RT-qPCR)

Total RNA was extracted from tissue samples using TRIzol reagent, followed by reverse transcription using TakaRa PrimeScript™ RT Master Mix. Real-time PCR was performed using AceQ Universal SYBR qPCR Master Mix. Gene expression levels were normalized to GAPDH and calculated via the 2^^−ΔΔCt^ method, with primer sequences provided in **Supplementary Table 1**.

#### 2.6.10 Western blot assay

Total proteins were extracted using RIPA buffer, separated by 10% SDS-PAGE, and transferred to PVDF membranes. After blocking with 5% skim milk and TBST washing, membranes were incubated overnight at 4 °C with the following primary antibodies: rabbit anti-Tnfsf12 (# ab 316929; Abcam), rabbit anti-Tnfrsf12a (# ab109365; Abcam), rabbit anti-p65 (# 8242; Cell Signaling Technology), rabbit anti-p-p65 (# 3033; Cell Signaling Technology), rabbit anti-IκBα (# 4812; Cell Signaling Technology), rabbit anti-p-IκBα (# 2859; Cell Signaling Technology). Following TBST washes, membranes were incubated with HRP-conjugated secondary antibodies at room temperature. Protein expression levels were normalized to GAPDH as the loading control.

#### 2.6.11 Immunohistochemical analysis

Tissue sections were processed through standard dehydration, paraffin embedding, and microtome sectioning (4 μm) prior to antigen retrieval. After blocking endogenous peroxidase activity, sections were incubated with primary antibodies against EP3 (1:500, GB111814, Servicebio) and EP4 (1:2000, GB113920, Servicebio) overnight at 4 °C, followed by PBS washes and incubation with HRP-conjugated secondary antibodies. Chromogenic detection was performed using DAB substrate, and counterstained sections were imaged under microscopy.

### 2.7 Correlation analysis and weighted gene co-expression network analysis

A Spearman rank correlation analysis was performed to evaluate pairwise associations among differential microbial taxa, metabolites, and genes, in order to explore the coordinated changes between gut microbiota, metabolic remodeling, and gene expression. Correlations were calculated based on group-level abundance or expression data, with correlation coefficients and their significance used to assess relationships across omics layers. Additionally, Mantel tests were conducted to examine the global relationships between duodenal low−grade inflammatory phenotypes—represented by pro−inflammatory cytokine levels (IL−6, IL−1β, and TNF−α)—and matrices of differential microbial composition, differential metabolite abundance, and transcriptomic expression profiles, systematically evaluating the association between inflammatory status and multi−omics features.

Upon completion of sample− and gene−level quality control of transcriptomic data, a weighted gene co−expression network analysis (WGCNA) was performed to construct gene co−expression networks. Based on gene expression similarity matrices, co−expression modules were identified using dynamic tree cut, and each module was represented by its module eigengene (ME). Group−level ME expression profiles were then generated by averaging MEs within each experimental group for subsequent multi−omics integration analysis. Spearman rank correlation was further employed to evaluate associations between MEs and differential metabolites, microbial taxa, and genes. Correlations were filtered based on statistical significance and correlation strength. Finally, an integrated multi−omics association network centered on MEs and incorporating differential genes, metabolites, and microbial taxa was constructed and visualized using Cytoscape, with key modules enhanced for clarity.

### 2.8 Statistical methods

GraphPad Prism (version 7) was used for statistical analysis and plotting, with a significance threshold of α=0.05 and *P < 0.05* considered statistically significant. All quantitative data are expressed as mean ± SD. Normality was assessed via the Shapiro–Wilk test and homogeneity of variance via Levene’s test. When both assumptions were met, a two-way ANOVA was applied to evaluate pre- and post-intervention differences. For between-group comparisons, one-way ANOVA followed by Tukey’s HSD was used. In the event of heteroscedasticity, Welch’s ANOVA with Tamhane’s T2 *post-hoc* was employed, and non-parametric analyses were used for non-normally distributed variables.

## 3 Results

### 3.1 EA enhances body weight and food intake

**Figure 2:**
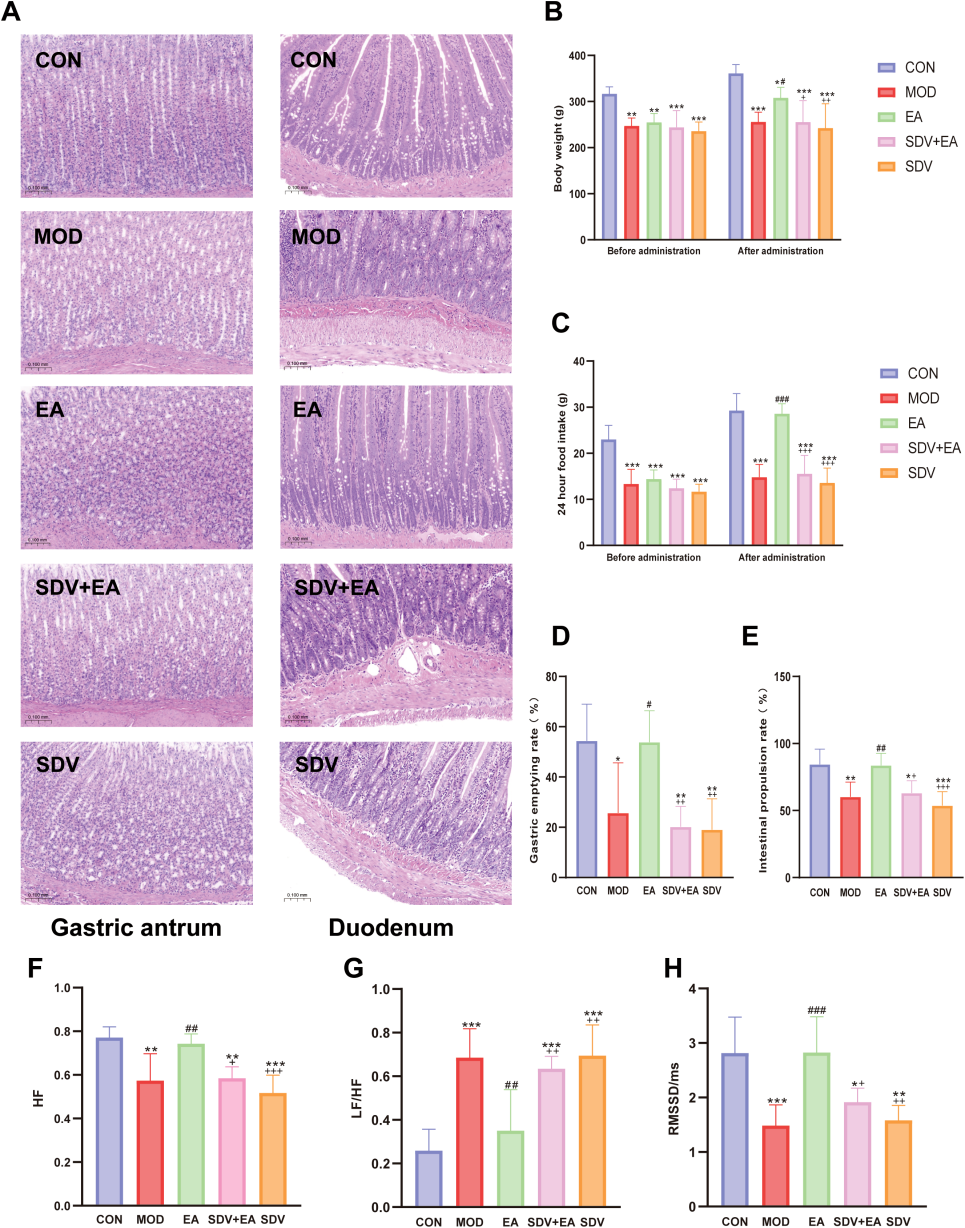
Phenotype of FD rats. **(A)** HE staining, **(B)** Weight, **(C)** 24-hour food intake, **(D)** Gastric emptying rate, **(E)** Intestinal propulsion rate, **(F)** HF, **(G)** LF/HF, **(H)** RMSSD. Statistical comparisons were made with the CON group, *P < 0.05, **P < 0.01, ***P < 0.001. Statistical comparisons were made with the MOD group, #P < 0.05, ##P < 0.01, ###P < 0.001. Statistical comparisons were made with the EA group, +P < 0.05, ++P < 0.01, +++ P < 0.001 (n=6rats/group).

HE staining revealed no significant structural damage in the gastric antrum or duodenum across groups (**Figure 2A**), confirming successful model establishment. FD model rats exhibited characteristic reductions in body weight and food intake, accompanied by gastric motility dysfunction. EA intervention significantly improved body weight (**Figure 2B**), enhanced 24-hour food intake (**Figure 2C**), and normalized gastrointestinal motility, as evidenced by increased gastric emptying rate and intestinal propulsion rate (**Figures 2D, E**). These therapeutic effects were attenuated following subdiaphragmatic vagotomy, indicating that the effects are mediated through vagal nerve pathways.

### 3.2 EA enhances vagal tone and restores sympathovagal balance

As shown in **Figures 2F–H**, the MOD exhibited significantly decreased HF (*P* < 0.01) and RMSSD (*P* < 0.001) along with elevated LF/HF ratio (*P* < 0.001) compared to CON. EA treatment effectively reversed these autonomic imbalances, increasing HF (*P* < 0.01) and RMSSD (*P* < 0.001) while reducing LF/HF ratio (*P* < 0.01). These therapeutic effects were abolished following vagotomy, which showed decreased HF power (*P* < 0.05) and RMSSD (*P* < 0.05), and increased LF/HF ratio (*P* < 0.01) compared to the EA group, demonstrating the critical role of vagal pathways in EA-mediated autonomic regulation.

### 3.3 EA improves gut microbiota

**Figure 3:**
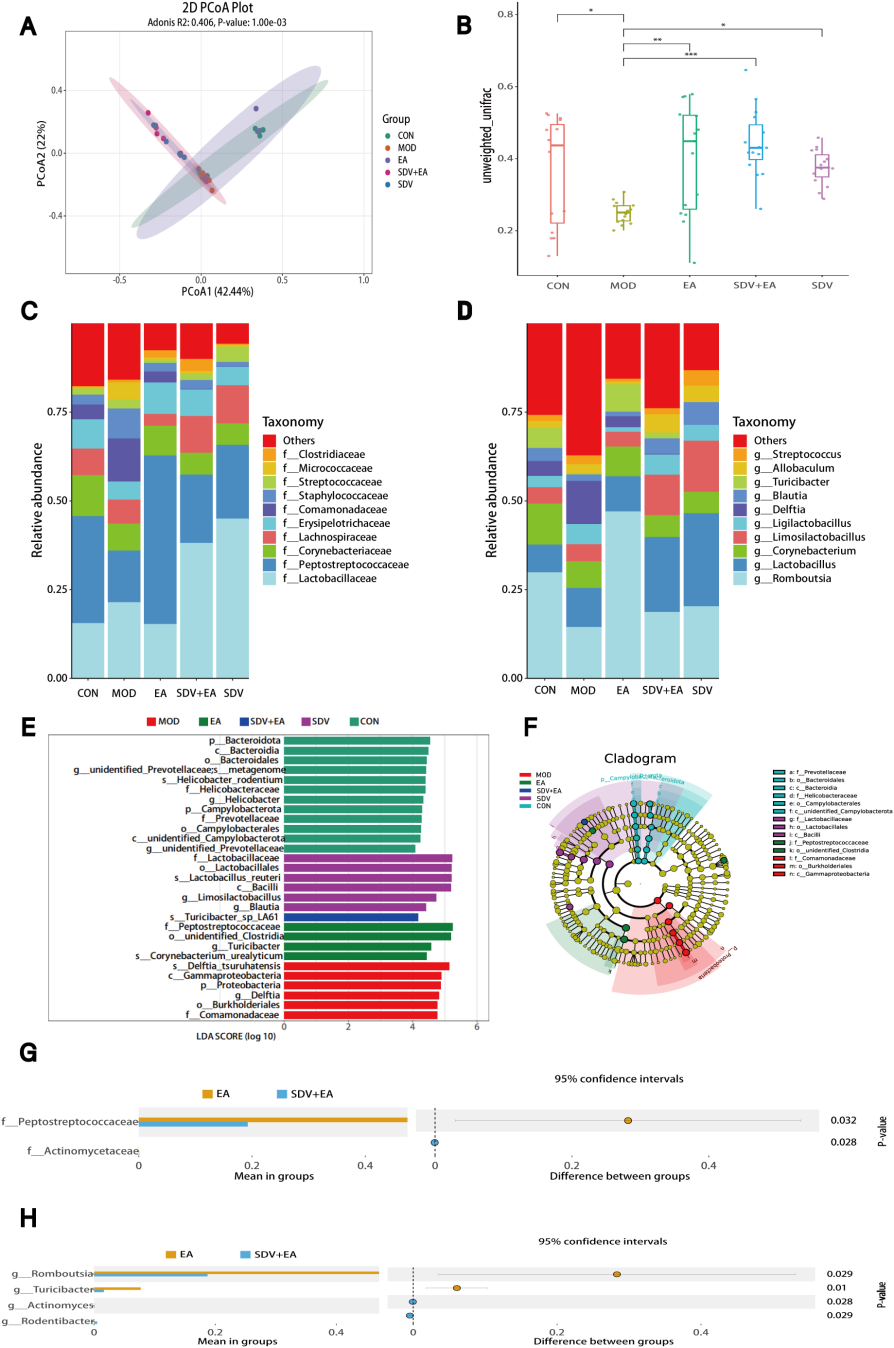
Duodenal microbial characteristics. **(A)** PCoA analysis, **(B)** Unweighted unifrac distance analysis, **(C)** Relative abundance of family level, **(D)** Relative abundance of genus level, **(E, F)** LEfSe analysis, **(G)** family-level and **(H)** genus-level comparisons revealed significant microbial differences between SDV+EA and SDV groups (n=6 rats/group). *P < 0.05, **P < 0.01, ***P < 0.001.

The rank-abundance and rarefaction curves demonstrated high sequencing depth and even species distribution (**Supplementary Figure 1**). α-diversity analysis revealed no significant differences between the CON and MOD groups. However, statistically significant differences were observed in Shannon and Simpson indices between the MOD and EA groups (*P* < 0.01), as well as in ACE and Chao1 indices between the EA and SDV+EA groups (*P* < 0.001), indicating that the EA-induced improvement in microbial richness and diversity was markedly attenuated following subdiaphragmatic vagotomy, thereby suggesting a critical role of the VN in mediating EA-driven microbiota modulation (**Supplementary Figure 1**). β-diversity analysis using PCoA revealed that the EA group exhibited a similar clustering pattern to the CON group, suggesting comparable gut microbiota composition, while the MOD, SDV, and SDV+EA groups displayed distinct distribution patterns (**Figure 3A**). OPLS-DA modeling further confirmed significant microbiota differences between MOD and CON groups, with EA intervention shifting the MOD microbial structure toward the CON profile (**Supplementary Figure 1**). Compared to CON, the MOD group showed significantly decreased unweighted unifrac distance (*P* < 0.05), which was remarkably increased after EA treatment (*P* < 0.01). However, vagotomy attenuated this effect, leading to reduced unweighted unifrac distance again (**Figure 3B**), highlighting the dependence of EA-mediated microbial restructuring on an intact VN.

At the family level, five major differentially abundant bacterial taxa were identified. Compared with the CON group, the MOD group exhibited increased relative abundances of *f_Lactobacillaceae* and *f_Streptococcaceae*, which were reversed by EA intervention but restored after vagotomy. Conversely, the MOD group showed decreased relative abundances of *f_Peptostreptococcaceae*, *f_Corynebacteriaceae*, and *f_Erysipelotrichaceae* compared to CON, with EA treatment restored their abundance levels - an effect that was similarly abolished by VN resection (**Figure 3C**). At the genus level, seven predominant bacterial taxa were identified. The MOD group demonstrated decreased relative abundances of *g_Romboutsia*, *g_Turicibacter*, and *g_Corynebacterium* compared to the CON group, which were restored by EA treatment but subsequently reduced again following vagotomy. Conversely, the relative abundances of *g_Lactobacillus*, *g_Ligilactobacillus*, *g_Allobaculum*, and *g_Streptococcus* were elevated in the MOD group relative to CON, with EA effectively reducing these levels - an effect that was reversed by vagotomy (**Figure 3D**). These findings indicate that EA-mediated normalization of microbial alterations in FD is largely dependent on VN integrity. Detailed information on differential microbial taxa is provided in **Supplementary Table 2**.

LEfSe analysis (LDA score >4) identified distinct bacterial taxa among the five groups (**Figures 3E, F**). Subsequent comparative analysis between EA and SDV+EA groups revealed significant differences at both family and genus levels, particularly in *f_Peptostreptococcaceae*, *g_Romboutsia*, *g_Turicibacter*, and *g_Allobaculum* (**Figures 3G, H**). Collectively, these findings demonstrate that EA partially restores gut microbiota structure in FD rats and that this restorative effect is predominantly mediated by vagal nerve pathways, as it is significantly attenuated by vagotomy.

### 3.4 EA regulates metabolism

**Figure 4:**
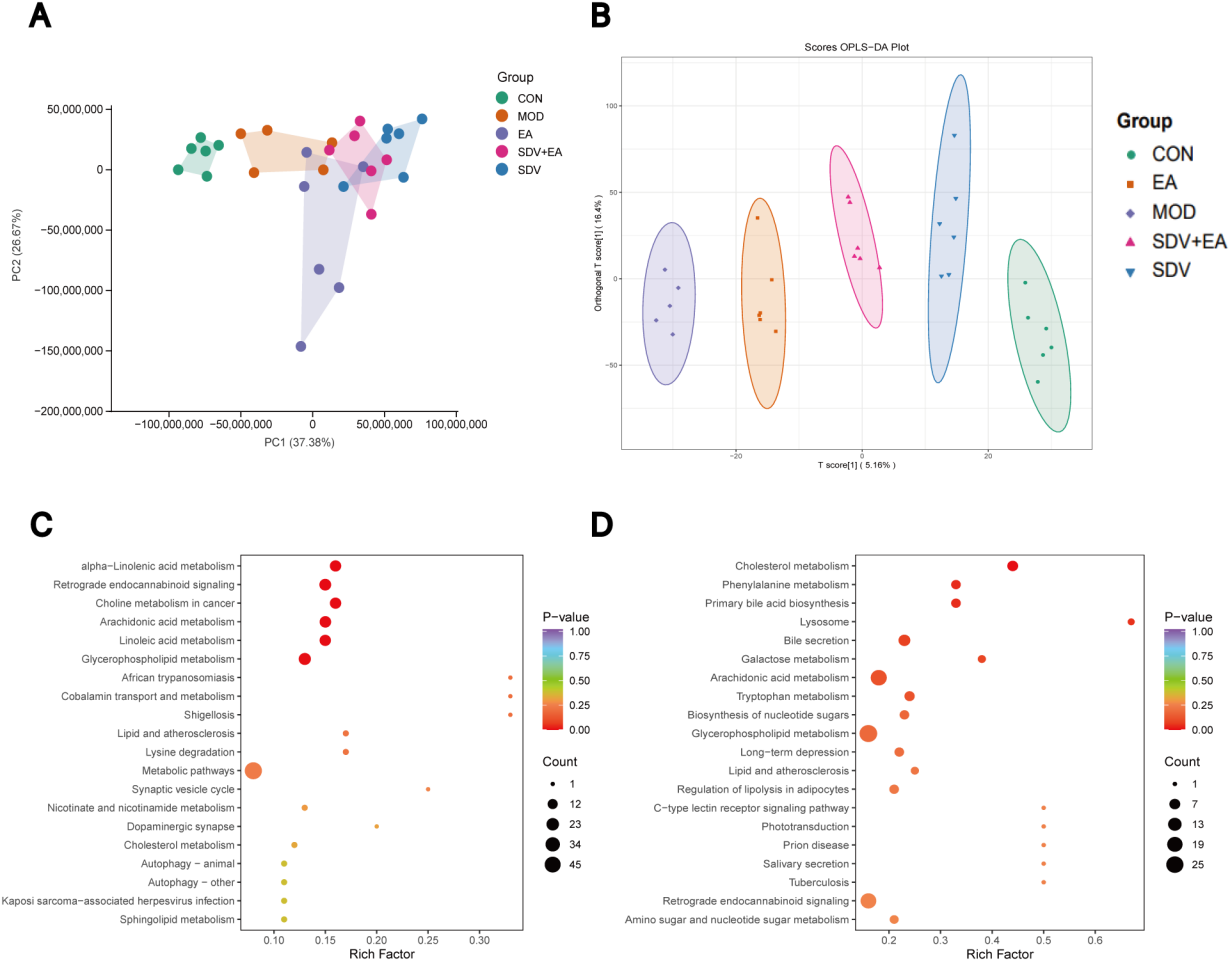
Duodenal content metabolomics results. **(A)** PCA analysis, **(B)** OPLS-DA analysis, **(C)** KEGG pathway analysis of differential metabolites comparing EA vs MOD groups, **(D)** KEGG pathway analysis of differential metabolites comparing EA vs SDV+EA groups (n=5 for MOD group, n=6 for all other groups).

Following quality control, one sample from the MOD group was excluded, leaving 29 samples for duodenal content metabolomics analysis. Quality control (QC) samples demonstrated well-shaped peaks and relatively uniform distribution in both positive and negative ion modes, as shown in total ion chromatograms (TIC) (**Supplementary Figure 2**). PCA revealed significant metabolic differences among the five groups (**Figure 4A**), while orthogonal partial least squares discriminant analysis (OPLS-DA) indicated good intra-group clustering and clear inter-group separation (**Figure 4B**).

A total of 24 differential metabolites were identified, including Cholesta-3,5-dien-7-one, Licofelone, Digoxigenin, 7-Hydroxymethotrexate, Hydroxymethylbilane, (1S,13S,18S)-12-[2-(4-hydroxyphenyl)ethyl]-18-methoxy-15-methyl-5,7-dioxa-12,15-diazapentacyclo[11.7.0.01,16.02,10.04,8]icosa-2,4(8),9,19-tetraen-14-one (C_26_H_28_N_2_O_5_), Trp-Phe-Glu, Leu-Asp-Leu, Ser-Leu-Val-Asn-Ala, Asn-Val-Glu-Glu, Asp-Gln-Phe-Arg, Met-Gln-His, Gly-Ala-Pro-Met-Phe-Val-NH2, Naphthyl glucuronide, Aspidin, ONO-8711, Toyocamycin, (1S,3Z,5R,7R)-3-[(3,4-dihydroxyphenyl)-hydroxy-methylene]-1-[(2S)-2-isopropenyl-5-methyl-hex-4-enyl]-6,6-dimethyl-5,7-bis(3-methylbut-2-enyl)bicyclo[3.3.1]nonane-2,4,9-trione (C_38_H_50_O_6_), TG(20:0/8:0/8:0), 5’-O-methylmelledonal, L-Naspa, Unii-WN080Z1OL0, gibberellin A3 O-beta-D-glucoside, and Amikacin hydrate. Comparative analysis revealed that all identified metabolites were downregulated in the MOD group compared to CON group. EA treatment significantly upregulated these metabolites, while subsequent vagotomy (after accounting for intrinsic vagotomy effects) caused their re-downregulation in the SDV+EA group relative to EA group. These findings indicate that the metabolic regulatory effects of EA are strongly dependent on intact vagal nerve signaling. Differential metabolites information is provided in **Supplementary Table 3**.

KEGG pathway analysis of the differential metabolites revealed significant enrichment in retrograde endocannabinoid signaling, arachidonic acid metabolism, glycerophospholipid metabolism, cholesterol metabolism, and lipid and atherosclerosis pathways when comparing both EA vs. MOD and EA vs. SDV+EA groups (**Figures 4C, D**; **Supplementary Figure 3**), suggesting these pathways as potential therapeutic targets for EA’s vagal nerve-mediated effects in FD.

### 3.5 EA regulates gene expression

**Figure 5:**
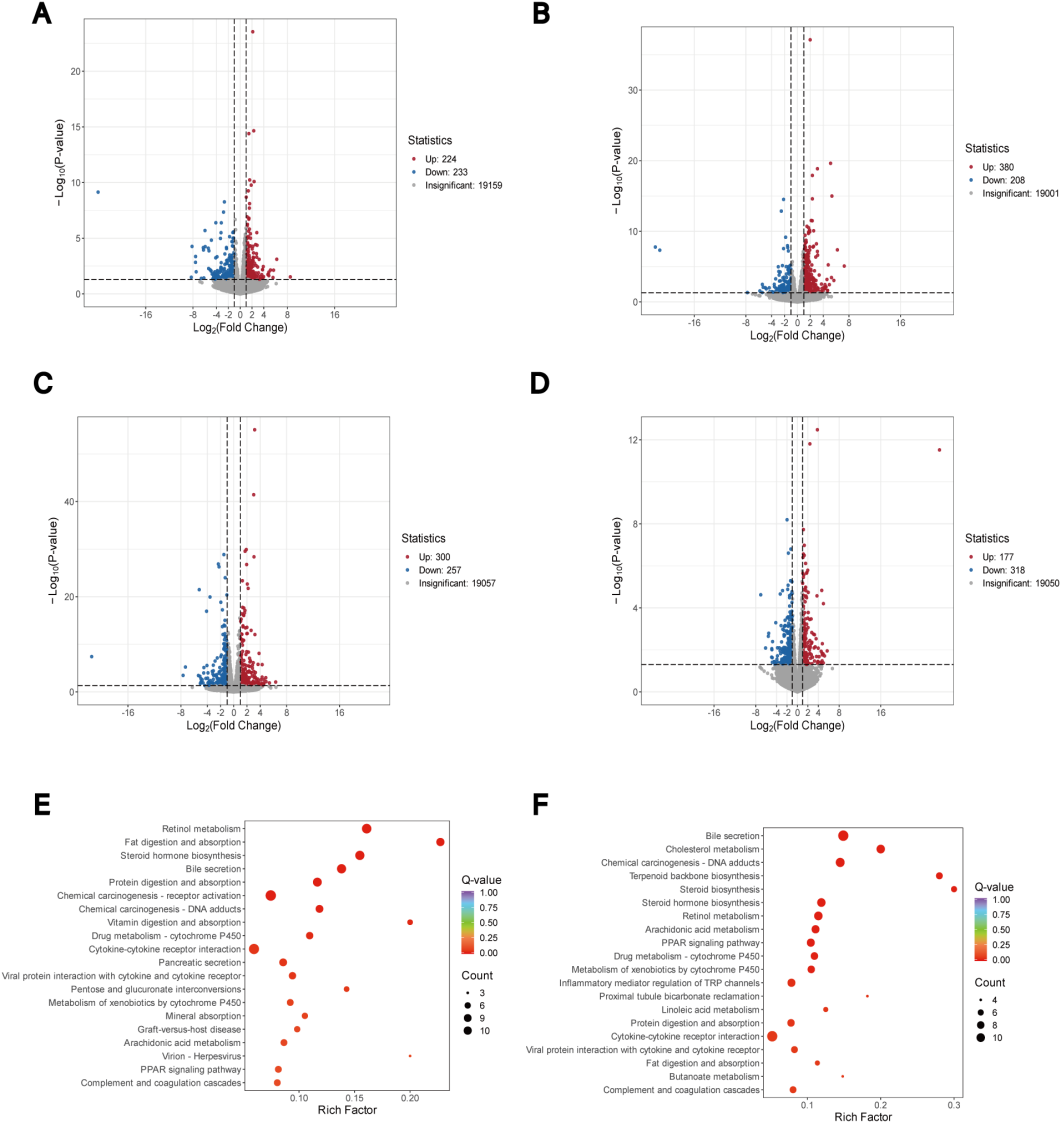
Duodenal transcriptomic results. **(A)** DESeq2 analysis of DEGs between CON and MOD groups, **(B)** MOD versus EA DEGs, **(C)** EA versus SDV+EA DEGs, and **(D)** MOD versus SDV DEGs, along with KEGG pathway analyses of **(E)** EA vs MOD and **(F)** EA vs SDV+EA comparisons (n=3 rats/group).

RNA sequencing analysis was performed on three randomly selected rats per group. Comparative analysis revealed 457 differentially expressed genes (DEGs) between MOD and CON groups (233 downregulated, 224 upregulated; **Figure 5A**). EA intervention resulted in 588 DEGs between EA and MOD groups (208 downregulated, 380 upregulated; **Figure 5B**), while vagotomy subsequently altered gene expression patterns, showing 557 DEGs between SDV+EA and EA groups (257 downregulated, 300 upregulated; **Figure 5C**). Integrated analysis of these DEGs, after controlling for the confounding effects of vagotomy itself (**Figure 5D**), identified 23 candidate genes (13 annotated and 10 unannotated) as potential mediators of EA’s therapeutic effects on FD through VN modulation. A focused analysis of these candidate genes revealed two distinct, VN-dependent regulatory patterns mediated by EA: (1) Nine genes - serine protease 22 (*Prss22*), LY6/PLAUR domain containing 3 (*Lypd3*), *AC093960.1*, TNF receptor superfamily member 12A (*Tnfrsf12a*), ATPase Na+/K+ transporting subunit alpha 3 (*Atp1a3*), vomeronasal 2 receptor 44 (*Vom2r44*), and Claudin 6 (*Cldn6*) - were significantly upregulated in the MOD group compared to CON group, downregulated following EA treatment, and subsequently re-upregulated after vagotomy; (2) Fourteen genes-including aryl-hydrocarbon receptor repressor (*Ahrr*), *RGD1566007*, complement factor D (*Cfd*), *AC109901.2*, *Cd209e*, and solute carrier family 4 member 10 (*Slc4a10*) - demonstrated the opposite pattern, showing downregulation in MOD, upregulation after EA, and re-downregulation following VN ablation (**Supplementary Table 4**). These bidirectional expression patterns consistently demonstrate that EA-induced transcriptional normalization in FD is largely contingent upon intact VN signaling. Among the identified DEGs, Tnfrsf12a emerged as a gene of particular interest, as a member of the TNF receptor superfamily critically involved in pro-inflammatory pathways and previously implicated in gastrointestinal inflammation. Additionally, we observed that *Homer3*, a gene involved in neuronal signaling transduction, was downregulated in the MOD group compared to CON group, upregulated following EA intervention, and subsequently downregulated again after vagotomy, although these changes did not reach statistical significance.

KEGG pathway analysis of DEGs revealed significant enrichment in fat digestion and absorption, steroid hormone biosynthesis, bile secretion, protein digestion and absorption, drug metabolism - cytochrome P450, cytokine-cytokine receptor interaction, arachidonic acid metabolism, PPAR signaling pathway, and complement and coagulation cascades in both MOD vs EA and EA vs SDV+EA comparisons (**Figures 5E, F**; **Supplementary Figure 4**). Importantly, the recurrence of these pathways in comparisons involving VN ablation indicates that EA-driven transcriptional regulation of metabolic and inflammatory pathways is VN-dependent. Integrated analysis of both transcriptomic and metabolomic data identified arachidonic acid metabolism as a potential key pathway mediating EA’s therapeutic effects on FD through VN regulation.

### 3.6 EA downregulates the expression of inflammatory cytokines

**Figure 6:**
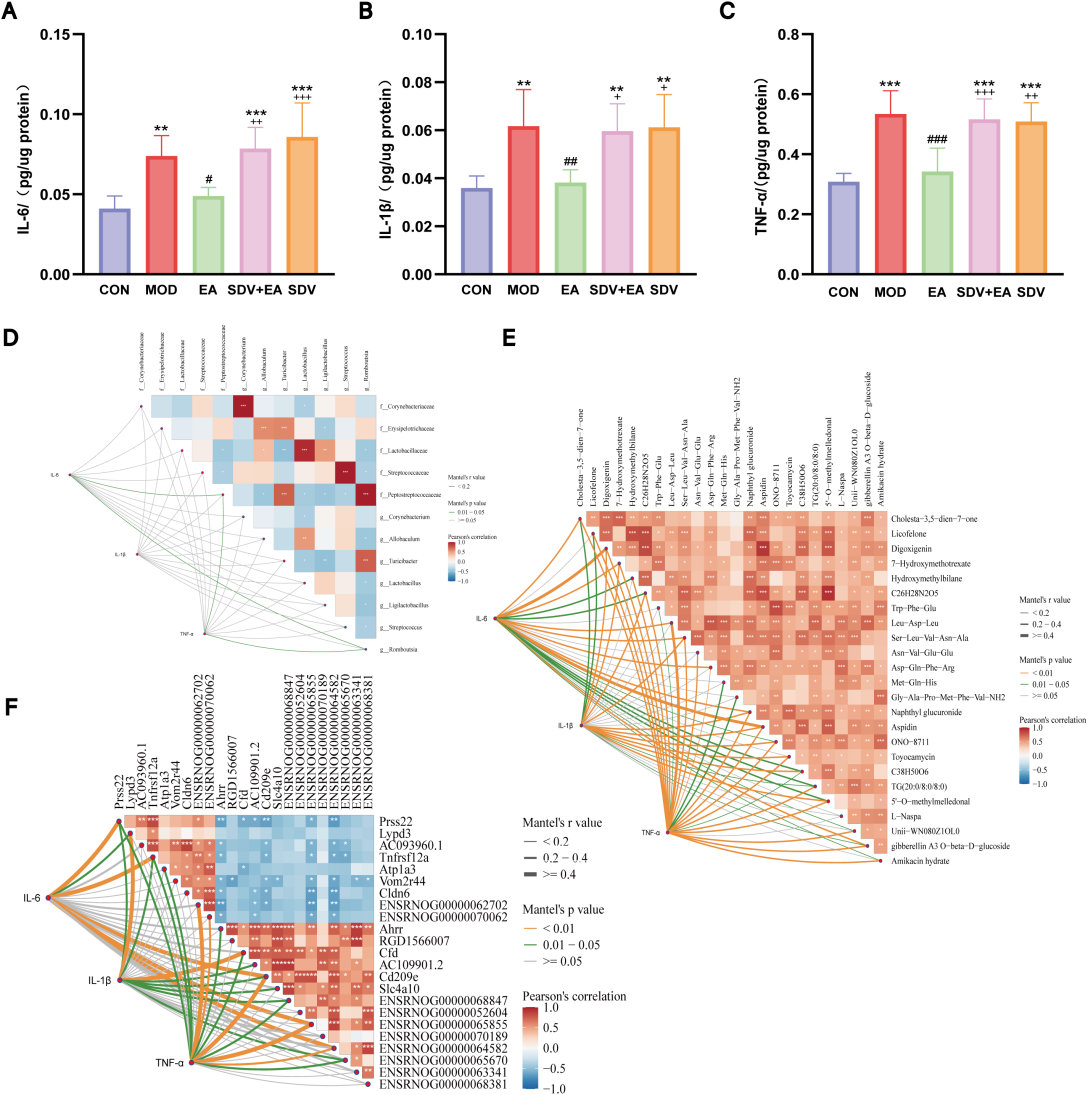
Pro-inflammatory cytokines and multi-omics correlations. **(A–C)** Duodenal levels of IL-6, IL-1b, and TNF-a measured by ELISA (n=6); **(D)** Correlation between pro-inflammatory cytokines and differential microbial taxa; **(E)** Correlation between pro-inflammatory cytokines and differential metabolites; **(F)** Correlation between pro-inflammatory cytokines and differentially expressed genes. *P < 0.05, **P < 0.01, ***P < 0.001. Statistical comparisons were made with the CON group, **P < 0.01, ***P < 0.001; Statistical comparisons were made with the MOD group, #P < 0.05, ##P < 0.01, ###P < 0.001; Statistical comparisons were made with the EA group, + P < 0.05, ++ P < 0.01, +++ P < 0.001.

Given that duodenal low-grade inflammation is a key pathological component of functional dyspepsia, characterized by elevated levels of pro-inflammatory cytokines (IL-6, IL-1β, TNF-α), and given that this inflammatory phenotype is closely associated with the differentially expressed gene Tnfrsf12a and the arachidonic acid metabolism pathway, we first systematically assessed the changes in these pro-inflammatory cytokines before further validating specific molecular pathways. Consistent with this pathological basis, the protein levels of IL-6, IL-1β, and TNF-α were significantly elevated in the MOD group compared to CON (*P* < 0.01, *P* < 0.01, *P* < 0.001). EA treatment markedly reduced their expression (*P* < 0.05, *P* < 0.01, *P* < 0.001), while subsequent vagotomy in the SDV+EA group reversed this anti-inflammatory effect, showing significant re-elevation versus EA group (*P* < 0.01, *P* < 0.05, *P* < 0.001) (**Figures 6A–C**), indicating VN-dependent mediation of EA’s anti-inflammatory action in FD.

### 3.7 Correlations analysis of inflammatory cytokines with the differential microbiome, metabolites, and genes

Mantel test analysis revealed IL-6 and TNF-α showed strong associations with the abundance of f_Peptostreptococcaceae and g_Romboutsia (**Figure 6D**). Notably, nearly all differential metabolites except L-Naspa exhibited significant relationships with inflammatory factors, with Cholesta−3,5−dien−7−one, Digoxigenin, 7−Hydroxymethotrexate, C_26_H_28_N_2_O_5_, Ser−Leu−Val−Asn−Ala, Naphthyl glucuronide, Aspidin, ONO-8711, C_38_H_50_O_6_, TG (20:0/8:0/8:0), 5’−O−methylmelledonal, and gibberellin A3 O−beta−D−glucoside demonstrating particularly strong correlations with all three cytokines examined (**Figure 6E**). In parallel, three genes—*Prss22*, *Tnfrsf12a*, and *Cd209e*—were strongly correlated with all three inflammatory cytokines (**Figure 6F**).

### 3.8 Correlations analysis of differential microbiota, differential metabolites, and differentially expressed genes

**Figure 7:**
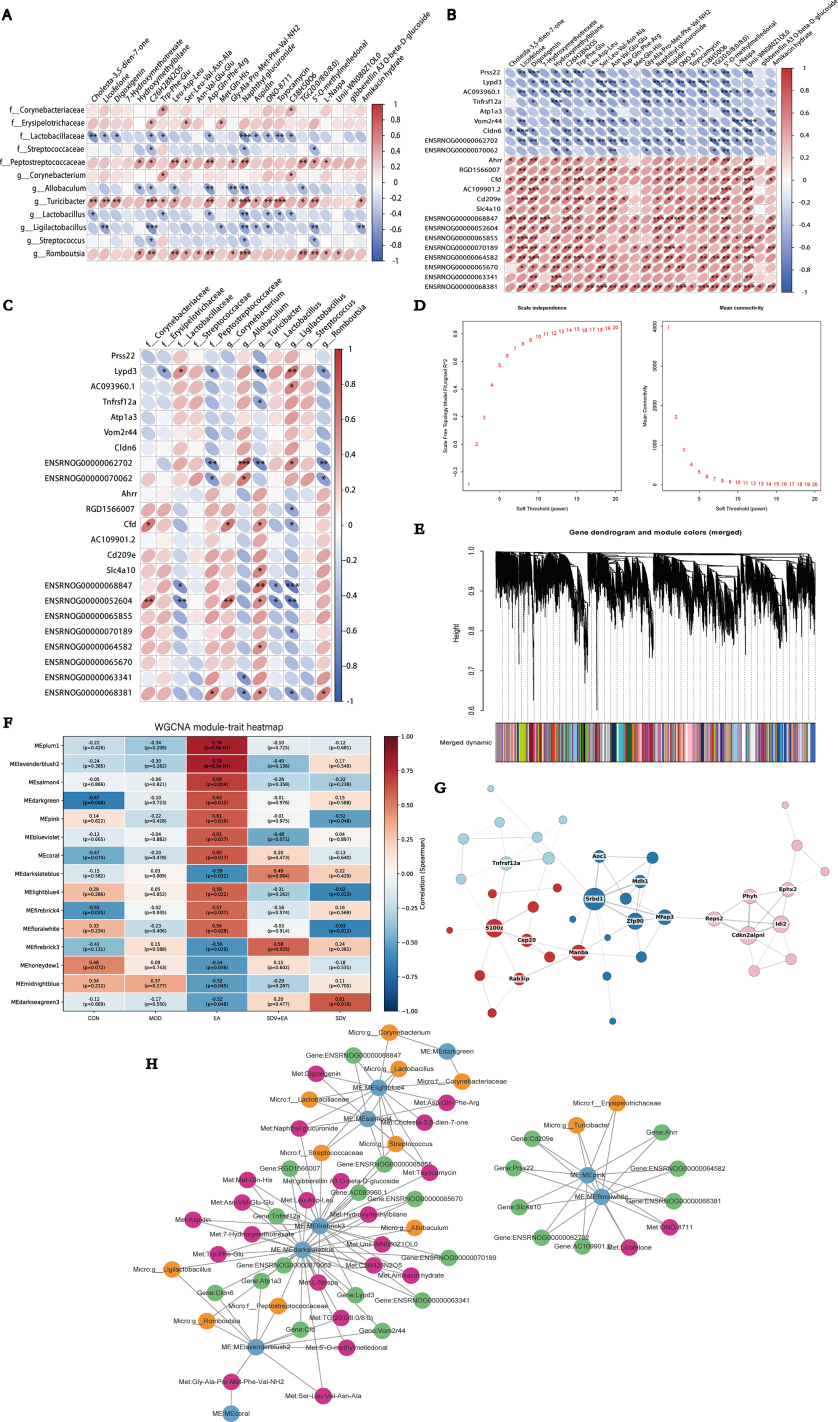
Correlation analysis, WGCNA and integrative multi-omics network analysis. **(A)** Correlation analysis between gut microbiota and differential metabolites; **(B)** Correlation analysis between differential metabolites and differentially expressed genes; **(C)** Correlation analysis between gut microbiota and differentially expressed genes; **(D)** Soft-threshold power selection for WGCNA; **(E)** Gene co-expression module identification by WGCNA; **(F)** Correlation heatmap between module eigengenes and experimental groups; **(G)** Hub gene analysis of the MEfirebrick3 module; **(H)** Integrated gene–metabolite–microbiota correlation network. Asterisks indicating statistical significance (*P < 0.05, **P < 0.01, ***P < 0.001).

Spearman correlation analysis revealed significant correlations among differential microbiota, metabolites, and genes: the relative abundance of g_Turicibacter showed positive correlations with metabolites C_26_H_28_N_2_O_5_, Naphthyl glucuronide, and Toyocamycin, while g_Romboutsia abundance similarly correlated positively with Naphthyl glucuronide. In contrast, f_Lactobacillaceae exhibited a significant negative correlation with Naphthyl glucuronide (**Figure 7A**). In addition, we found that the key differential gene Tnfrsf12a was significantly negatively correlated with metabolites such as Hydroxymethylbilane, Gly-Ala-Pro-Met-Phe-Val-NH_2_, and 5’-O-methylmelledonal, as well as with g_Turicibacter (**Figures 7B, C**). The correlation analyses among differential microbiota, differential metabolites, and differential genes are presented in **Supplementary Tables 5**-**7**.

### 3.9 WGCNA identifies key gene co-expression modules and a multi-omics interaction network

To elucidate how EA modulates the transcriptional network underlying the duodenum of FD, we performed WGCNA on quality−controlled transcriptomic data. A soft−thresholding power of 10 was selected to achieve scale−free topology. Dynamic tree cutting identified co−expression modules (minModuleSize=30, deepSplit=2), yielding several robust modules (**Figures 7D, E**). Correlation analysis between MEs and experimental groups revealed that MEfirebrick3 was negatively correlated with EA treatment (cor = −0.56), and positively correlated again after vagotomy (SDV+EA, cor = 0.58) (**Figure 7F**). These results indicate that aberrant activation of MEfirebrick3 is associated with FD, is suppressed by EA, and that this regulatory effect is attenuated after vagotomy, suggesting that EA’s modulation of this inflammation−related transcriptional module depends on intact vagal signaling. Further analysis of MEfirebrick3 revealed enrichment of genes involved in inflammatory and immune responses. Notably, *Tnfrsf12a*, a member of the TNF receptor superfamily, occupied a central position within the module (**Figure 7G**), implying its potential key role in mediating EA−induced, vagus−dependent regulation of duodenal inflammation.

The multi−omics network analysis revealed correlations among duodenal microbiota, metabolites, and genes across different modules, which are regulated by EA via the vagus nerve. Within this network, *Tnfrsf12a* was connected to metabolites—including Unii-WN080Z1OL0, Trp-Phe-Glu, Toyocamycin, TG(20:0/8:0/8:0), and Ser-Leu-Val-Asn-Ala—through two modules (MEdarkslateblue and MEfirebrick3) that showed a pronounced negative correlation with EA effects. *Tnfrsf12a* was also linked to microbial taxa such as *f_Streptococcaceae* and *g_Allobaculum* (**Figure 7H**). The key metabolic directions highlighted by this multi-omics network align with the significantly enriched linoleic acid metabolism pathway previously identified by KEGG enrichment analysis, further supporting the potential pivotal role of *Tnfrsf12a* and its co−expression module in EA−mediated regulation of duodenal inflammation.

### 3.10 EA inhibits the TWEAK/Fn14/NF-κB signaling pathway

**Figure 8:**
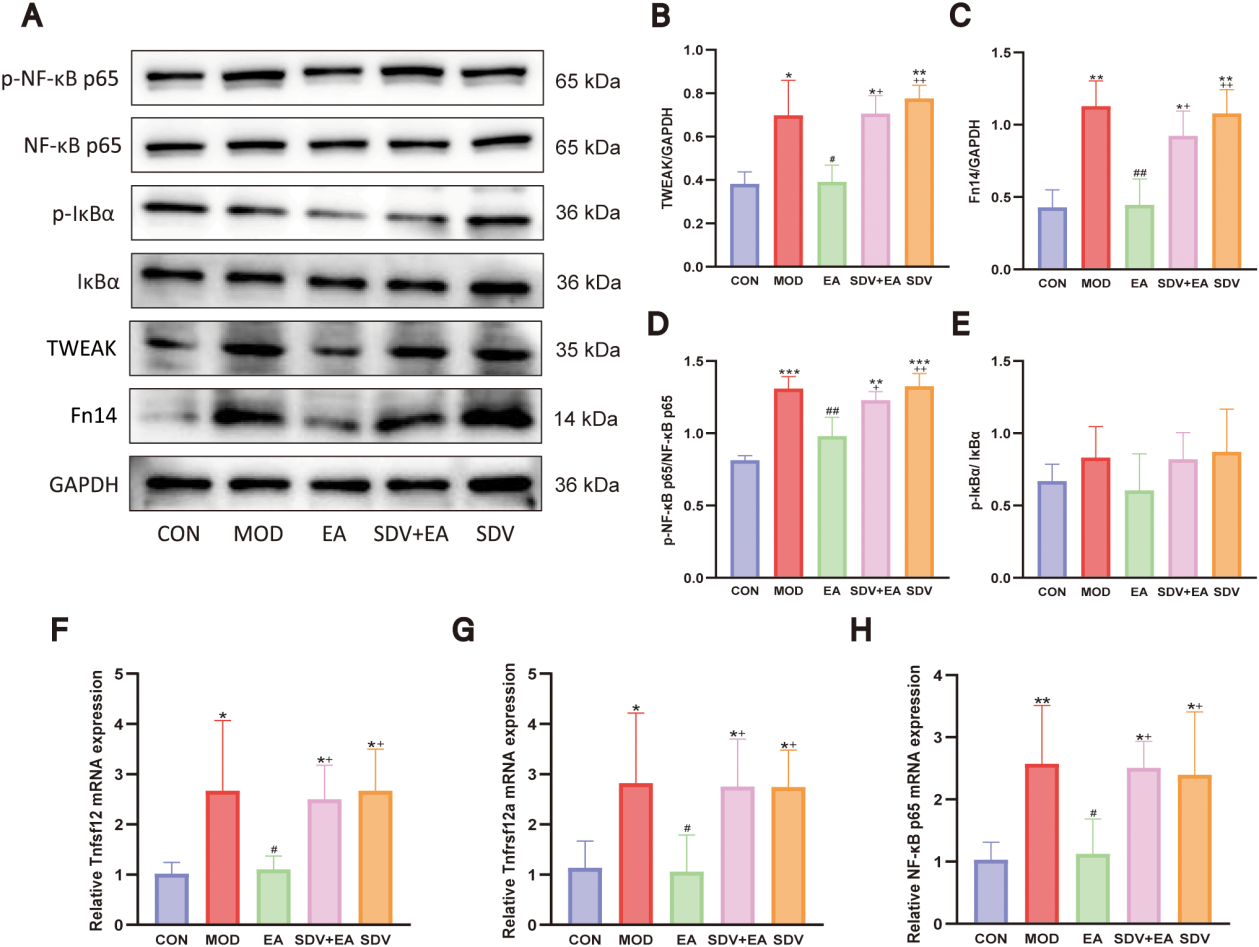
EA’s effects on the TWEAK/Fn14/NF-κB pathway of FD model rats. **(A–E)** Western blot analysis of pathway-related proteins (TWEAK, Fn14, p-NF-κB p65/NF-κB p65, and p-IκBα/IκBα; n=3) and **(F–H)** RT-qPCR quantification of Tnfsf12, Tnfrsf12a, and NF-κB p65 mRNA expression (n=6). Statistical comparisons were made with the CON group, *P < 0.05, **P < 0.01, ***P < 0.001; Statistical comparisons were made with the MOD group, #P < 0.05, ##P < 0.01; Statistical comparisons were made with the EA group, + P < 0.05, ++ P < 0.01.

The gene *Tnfrsf12a* encodes fibroblast growth factor inducible 14 (Fn14), while its ligand TNF-like weak inducer of apoptosis (TWEAK) is encoded by *Tnfsf12*. The TWEAK/Fn14 axis constitutes a crucial signaling pathway that modulates inflammatory and tissue damage responses (30). Western blot analysis revealed that the MOD group exhibited significantly elevated expression of TWEAK, Fn14, and p-NF-κB p65/NF-κB p65 ratio (*P* < 0.05, *P* < 0.01, *P* < 0.001, respectively) compared to CON group, indicating NF-κB pathway activation. EA treatment significantly reduced these markers (*P* < 0.05, *P* < 0.01, *P* < 0.01), an effect that was reversed by vagotomy. Although p-IκBα/IκBα showed similar modulation trends, the intergroup differences did not reach statistical significance (**Figures 8A–E**).

RT-qPCR was used to validate the regulatory effect of EA, mediated via the VN, on the mRNA expression of the receptor gene *Tnfrsf12a* (encoding Fn14), its ligand gene *Tnfsf12* (encoding TWEAK), and NF-κB p65. The results demonstrated that MOD induction significantly upregulated mRNA expression of Tnfsf12, Tnfrsf12a, and NF-κB p65 (*P* < 0.05, *P* < 0.05, *P* < 0.01, respectively) compared to CON group, while EA treatment effectively downregulated these transcripts (*P* < 0.05 for all). This therapeutic effect was significantly attenuated following vagotomy (*P* < 0.05 for all comparisons) (**Figures 8F–H**). Collectively, these results indicate that the TWEAK/Fn14/NF-κB signaling pathway plays a critical role in FD pathogenesis, and that EA inhibits this pro-inflammatory pathway predominantly through VN-dependent mechanisms, highlighting the VN as a key mediator linking EA stimulation to molecular inflammatory regulation in the duodenum.

### 3.11 EA suppresses the arachidonic acid metabolism pathway

**Figure 9:**
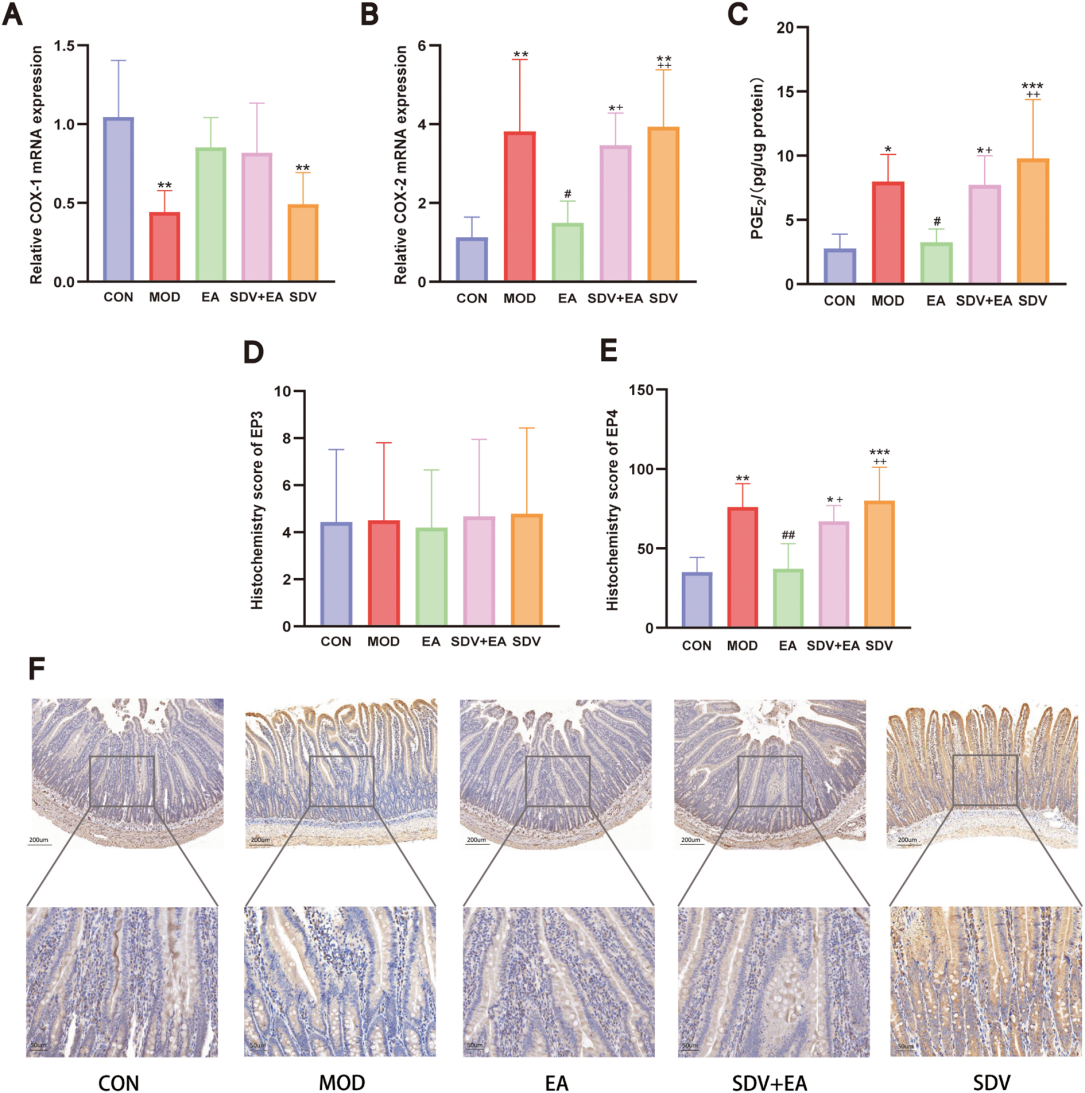
EA’s effects on the arachidonic acid metabolic pathway in FD rats. **(A, B)** mRNA levels of COX-1, COX-2 (n=6); **(C)** PGE_2_ level by ELISA (n=6); **(D)** EP3 receptor immunohistochemistry (n=5); **(E, F)** EP4 receptor immunohistochemistry (n=5). Statistical comparisons were made with the CON group, *P < 0.05, **P < 0.01, ***P < 0.001; Statistical comparisons were made with the MOD group, #P < 0.05; Statistical comparisons were made with the EA group, + P < 0.05, ++ P < 0.01.

RT-qPCR analysis of duodenal tissues revealed significant modulation of arachidonic acid metabolic pathway components. COX-1 mRNA expression was markedly decreased in the MOD group compared to CON (*P* < 0.01), with non-significant elevation after EA treatment (*P*>0.05) that was unaffected by vagotomy (*P*>0.05). Conversely, COX-2 mRNA expression was dramatically upregulated in MOD (*P* < 0.01), significantly downregulated by EA (*P* < 0.05), and re-elevated following vagotomy (*P* < 0.05) (**Figures 9A, B**).

The expression levels of PGE_2_ in the duodenum of rats from each group were compared using the ELISA method. The results revealed that compared with the CON group, PGE_2_ expression was significantly increased in the MOD group (*P* < 0.05). Following electroacupuncture intervention, PGE_2_ expression was significantly decreased (*P* < 0.05), whereas vagotomy led to a significant increase in PGE_2_ expression (*P* < 0.05; **Figure 9C**).

Immunohistochemical analysis of PGE_2_ downstream receptors revealed distinct regulatory patterns: while EP3 expression showed a non-significant change in the MOD group compared to CON group, EP4 expression was significantly increased in MOD (*P* < 0.01), reduced by EA treatment (*P* < 0.01), and subsequently upregulated again following vagotomy (*P* < 0.05) (**Figures 9D–F**). Overall, the data confirm that EA exerts an inhibitory effect on the arachidonic acid metabolic cascade in FD, with key components of this suppression being reversed upon vagotomy, thereby highlighting the essential role of vagal neurotransmission.

## 4 Discussion

FD clinically manifests as epigastric pain, bloating, early satiety, appetite loss, and belching, with its pathological hallmark being the absence of identifiable organic lesions. Evidence indicates that impaired brain-gut axis regulation, particularly involving central and peripheral neural dysregulation, represents a key pathophysiological mechanism, where reduced vagal tone is considered a critical pathogenic factor (7). Duodenal low−grade inflammation serves as the core peripheral pathological basis of FD, contributing to brain−gut axis dysregulation by activating sensory nerve endings, amplifying inflammatory signaling, and disrupting gastrointestinal motility regulation. EA serves as an effective central-peripheral modulation therapy, demonstrating multi-target regulatory effects on FD and other DGBIs through multiple pathways (14, 31). This study employed a multi-omics approach to systematically investigate the therapeutic mechanisms of EA in FD through VN modulation, integrating analyses of gut microbiota composition, transcriptomic profiles, and metabolic alterations. We demonstrated for the first time that EA ameliorated FD by VN-dependent suppression of both TWEAK/Fn14/NF-κB signaling and arachidonic acid metabolism pathways, thereby alleviating duodenal low−grade inflammation, reducing levels of pro−inflammatory factors such as TNF−α, IL−1β, IL−6, and PGE_2_, and concurrently improving gut microbial dysbiosis and metabolic disturbances in FD rats. These findings established an innovative neuro-immune-metabolic-microbial network underlying EA’s therapeutic effects in FD pathogenesis.

IA, a protease inhibitor that inactivates enzymatic activity without causing protein denaturation, induces cellular hypoxia, mucosal injury, and anxiety/depression-like behaviors, making it an established model compound for studying the pathophysiological mechanisms of digestive disorders including chronic gastritis and FD (32). The tail-clamping stress method effectively simulates emotional triggers of FD by provoking anxiety and tension in rodents (33). In this study, we successfully established a FD rat model by combining gavage of 0.2 mL of 0.1% IA with 2% sucrose solution and tail-clamping stress, effectively mimicking the brain-gut interaction dysfunction observed in FD patients. The model validation included characteristic phenotypic changes (reduced body weight, decreased food intake, and unkempt fur), absence of significant histopathological damage in gastric and duodenal tissues by HE staining, and HRV-confirmed autonomic imbalance with sympathetic-vagal dysregulation, collectively demonstrating successful model induction.

Acupuncture has been practiced in China for millennia, with EA representing a modern adaptation that replaces manual stimulation with electrical current. The ST36 (Zusanli) and ST37 (Shangjuxu) acupoints are critically important for treating gastrointestinal disorders. EA at ST36 enhances gastric slow-wave frequency and propagation velocity in FD patients, improves regularity of gastric myoelectrical activity, increases preprandial gastrin levels, accelerates gastric emptying, and alleviates both dyspeptic symptoms and psychological comorbidities (anxiety/depression) (34–36). Animal studies confirm that EA at ST36 intervention restores gastric slow-wave rhythm and autonomic nervous system function in FD models (14). The ST37 acupoint is widely utilized in treating various gastrointestinal disorders, including irritable bowel syndrome (IBS) (37, 38), constipation (39), and post-operative colorectal cancer conditions (40), demonstrating significant efficacy in regulating gastrointestinal motility and ameliorating inflammatory states. Combined application of ST36 and ST37 has been shown to effectively alleviate visceral hypersensitivity in post-inflammatory functional gastrointestinal disorders (41). Mechanistically, the therapeutic effects of stimulating ST36 and ST37 may converge on the modulation of the vagus nerve. A study demonstrated that EA at these acupoints significantly enhances gastric electrical activity and increases neuronal firing within the dorsal vagal complex (nucleus tractus solitarius and dorsal motor nucleus of the vagus), effects that are abolished following subdiaphragmatic vagotomy (24). With regard to stimulation parameters, dense–disperse (2/100 Hz) EA was adopted to reduce acupuncture sensation habituation, and its therapeutic efficacy has been well documented in previous studies of gastrointestinal disorders (25, 42). These findings collectively establish acupuncture at ST36 and ST37 as a safe and effective therapeutic approach for digestive system pathologies. Therefore, this study selected EA at the ST36 and ST37 acupoints, using a dense–disperse stimulation pattern (2/100 Hz), to evaluate its therapeutic efficacy in FD model rats and investigate the underlying mechanisms, aiming to provide more robust scientific evidence for clinical applications.

The VN, a mixed nerve composed of 80% afferent (sensory) and 20% efferent (motor) fibers, plays a pivotal role in gut-brain communication. Afferent fibers transmit gastrointestinal signals to the nucleus tractus solitarius (NTS) in the medulla, which subsequently relays visceral information to the dorsal motor nucleus (DMV) to modulate efferent function through vagal reflexes. This vagal circuitry, encompassing both brainstem pathways and efferent fibers, regulates gastric activity via dual neurotransmitter release: excitatory acetylcholine (enhancing motility) and inhibitory non-adrenergic non-cholinergic (NANC) transmitters (suppressing motility) through post-synaptic membrane receptors (23, 43). Thus, the VN serves as a critical bidirectional interface between the central nervous system and gastrointestinal tract. HRV is a key biomarker for assessing autonomic nervous system function, with the LF (0.07-0.8 Hz) component primarily reflecting sympathetic activity and the HF (0.8-4.0 Hz) component representing VN modulation. The LF/HF ratio and RMSSD are established indicators of sympathovagal balance (44). Previous studies have demonstrated that FD is characterized by autonomic dysfunction, specifically increased sympathetic activity and reduced vagal tone (9, 10, 12–15, 17, 18). Consistent with these findings, our study observed elevated LF/HF ratios in FD rats, indicating sympathetic hyperactivity coupled with vagal suppression. EA intervention significantly decreased the LF/HF ratio while increasing HF, demonstrating effective restoration of sympathovagal balance.

To further elucidate the mechanistic involvement of the VN in FD pathogenesis, we investigated the therapeutic outcomes of EA following vagotomy. The results demonstrated that surgical ablation of the VN completely reversed EA’s beneficial effects on multiple FD parameters, including food intake, body weight gain, gastrointestinal motility, and restoration of sympathovagal balance. These findings are consistent with previous reports that establish the critical role of vagal integrity in EA-mediated gastrointestinal regulation. While current research primarily focuses on phenomenological observations and monitoring of VN activity in FD (13, 14, 18, 45), the downstream molecular mechanisms and signaling pathways remain poorly characterized. To address this knowledge gap, our study innovatively integrated multi-omics analyses with experimental validation of key targets, thereby elucidating the fundamental mechanisms through which EA modulates VN function to ameliorate FD.

Our study demonstrated that EA modulated the composition of gut microbiota in FD rats through VN regulation, highlighting the critical role of microbial communities in FD pathogenesis. Taxonomic analysis identified five VN-associated bacterial families (*f_Lactobacillaceae*, *f_Streptococcaceae*, *f_Peptostreptococcaceae*, *f_Corynebacteriaceae*, *f_Erysipelotrichaceae*) and seven differentially abundant genera (*g_Romboutsia*, *g_Turicibacter*, *g_Corynebacterium*, *g_Lactobacillus*, *g_Ligilactobacillus*, *g_Allobaculum*, *g_Streptococcus*) that were significantly altered by EA intervention. Among these 12 microbial taxa, seven Firmicutes members-including *f_Lactobacillaceae*, *f_Streptococcaceae*, *f_Peptostreptococcaceae*, *g_Lactobacillus*, *g_Ligilactobacillus*, *g_Allobaculum*, and *g_Streptococcus*-play significant roles in gastrointestinal pathophysiology, particularly in digestive disorders. The *Allobaculum* activates intestinal epithelial cells in murine models and is closely associated with inflammatory bowel disease (IBD) pathogenesis (46). Streptococcus as opportunistic pathogens, predominantly cause gastrointestinal and respiratory disorders through fibronectin-binding surface adhesins and lipoteichoic acid-mediated host cell adhesion, while utilizing M protein to evade macrophage phagocytosis (47). Recent study reveals that Streptococcus anginosus, beyond Helicobacter pylori, contributes to atrophic gastritis and gastric carcinogenesis through TMPC surface protein interaction with gastric epithelial ANXA2 receptors, subsequently activating MAPK signaling pathways (48). Our study observed an unexpected elevation of *g_Lactobacillus* and *g_Ligilactobacillus* in FD rats, which contrasts with conventional understanding, while current evidence establishes that lactobacilli generally exert probiotic effects including microbiota modulation, pathogen exclusion, immune enhancement, and anti-carcinogenesis (49) through mechanisms such as promoting mucin/IgA secretion and inhibiting Helicobacter pylori adhesion (50). Our results demonstrated that IA intervention unexpectedly increased lactobacilli abundance in the duodenal contents of FD model rats. Similar positive correlations between lactobacilli proliferation and disease progression were observed in patients with IBD and IBS (51–53), as well as in systemic autoimmune disorders (54). We propose that lactobacilli exhibit significant functional complexity and diversity, with a substantial proportion of strains currently under investigation for their context-dependent roles in digestive disorders (55).

We systematically analyzed metabolic alterations in duodenal contents of FD rats following EA and vagotomy-combined EA interventions. Cross-comparison of differential metabolites across groups (CON vs. MOD, MOD vs. EA, EA vs. SDV+EA, SDV+EA vs. SDV) identified 24 VN-mediated metabolic targets of EA. We first focused on the metabolic changes closely related to FD, among which the metabolism of various amino acids such as Trp-Phe-Glu, Met-Gln-His, Leu-Asp-Leu, Gly-Ala-Pro-Met-Phe-Val-NH2 are closely related to FD. The correlation analysis results showed that the above amino acids were significantly correlated with the expression levels of IL-6, IL-1 β, and TNF - α. Glutamate serves as a crucial excitatory neurotransmitter that shows elevated levels in the somatosensory cortex (SSC) of FD patients, where it correlates with postprandial distress and anxiety, likely through visceral hypersensitivity (56). In the gastrointestinal tract, it functions as a versatile amino acid involved in taste perception, metabolism, and energy production (57). Glutamatergic signaling also mediates visceral pain in IBS (58), suggesting glutamate dysregulation may represent a common pathological mechanism across FGIDs, including FD. Licofelone is a dual 5-LOX/COX inhibitor that blocks both COX-1 and COX-2, reducing PGE_2_ production and improving acetic acid-induced colitis (59). ONO-8711 acts as a selective EP1 receptor antagonist, inhibiting PGE_2_ binding to improve visceral hypersensitivity and induce apoptosis, with therapeutic potential in colon cancer (60), hepatocellular carcinoma (61), and breast cancer (62). Both metabolites suppress arachidonic acid metabolism through distinct mechanisms. Our findings confirmed that EA upregulated both Licofelone and ONO-8711, consistent with their known therapeutic benefits, thereby providing experimental support for FD treatment. Correlation analysis further confirmed that both ONO−8711 and Licofelone were strongly associated with the levels of inflammatory cytokines, including IL−6, IL−1β, and TNF−α. Toyocamycin binds to the CDK9 catalytic site through its unique conformation, inducing GFP reactivation and suppressing clonogenic capacity in human colon cancer cells (63). It also demonstrates significant antitumor potential by modulating multiple apoptotic pathways (64, 65). Similarly, aspidin and its derivative aspidin PB exhibit therapeutic effects against liver cancer (66) and osteosarcoma (67) through apoptosis regulation. Naphthyl glucuronide serves as a clinical biomarker for β-glucuronidase activity, where elevated enzyme levels indicate hepatic or urinary disorders. In the gastrointestinal tract, microbial-derived β-glucuronidase plays a critical role in digestive diseases, with reduced β-glucuronidase activity in IBD patients linked to impaired intestinal barrier function and inflammatory responses (68). Our results demonstrated that Naphthyl glucuronide levels significantly correlated with pro-inflammatory cytokines (IL-6, IL-1β, TNF-α), further supporting this association. Digoxin, a cardiac glycoside known to cause nonspecific gastrointestinal symptoms (e.g., anorexia, nausea, vomiting), exhibits protective effects in nonalcoholic steatohepatitis by inhibiting PKM2-dependent HIF-1α activity, thereby reducing oxidative stress and inflammation while ameliorating high-fat diet-induced liver injury, steatosis, and hepatitis (69). We also identified amikacin hydrate, the hydrated form of amikacin used primarily against Gram-negative bacterial infections (70), suggesting potential links between infectious diseases and FD-related complications. Correlation analyses further confirmed associations between amikacin and inflammatory cytokine expression. The mechanistic roles of cholesta-3,5-dien-7-one, gibberellin A3 O-beta-D-glucoside, 5’-O-methylmelledonal, and hydroxymethylbilane in digestive disorders remain to be elucidated. KEGG pathway analysis of the differential metabolites revealed significant enrichment in metabolism-related pathways, primarily arachidonic acid metabolism, glycerophospholipid metabolism, and cholesterol metabolism.

We identified 23 key genes mediating EA’s therapeutic effects on FD through VN modulation, including 13 annotated targets: *Prss22*, *Lypd3*, *AC093960.1*, *Tnfrsf12a*, *Atp1a3*, *Vom2r44*, *Cldn6*, *Ahrr*, *RGD1566007*, *Cfd*, *AC109901.2*, *Cd209e*, and *Slc4a10*. This study provides novel insights by identifying candidate genes potentially contributing to FD pathogenesis. While some genes have established roles in diverse diseases and metabolic pathways, their direct involvement in FD remains incompletely characterized. These findings not only expand current knowledge but also establish a new theoretical framework for investigating FD’s molecular mechanisms. *Prss22* encodes a serine protease involved in proteolysis, signal transduction, and immunomodulation, showing elevated expression in breast cancer linked to lymph node metastasis and poor prognosis (71). *Lypd3* demonstrates overexpression in colorectal (72) and lung cancers (73), where it enhances tumor cell migration through integrin signaling regulation, suggesting potential as both a biomarker and therapeutic target. *Cldn6* encodes a tight junction protein that regulates epithelial/endothelial barrier function and selective permeability. As a Claudin family member, it exhibits dual oncogenic roles: suppressing colon cancer progression via TYK2/STAT3 signaling (74) and inhibiting breast cancer through JNK/c-Jun-mediated autophagy regulation (75), while promoting hepatocellular carcinoma via E-cadherin/N-cadherin/vimentin modulation (76). Its tumor-specific expression and functional versatility make Cldn6 an ideal therapeutic target (77). *Atp1a3* encodes the α3 subunit of Na^+^/K^+^-ATPase, a critical regulator of membrane potential and ionic homeostasis strongly associated with neurological disorders including cerebellar ataxia (78) and epilepsy (79). *Ahrr* functions as a negative regulator of the aryl hydrocarbon receptor (AhR) signaling pathway, playing key roles in cell growth/differentiation and contributing to various cancers such as gallbladder (80), lung (81), and ovarian cancers (82). *Cfd* encodes a pivotal serine protease in the alternative complement pathway that cleaves Factor B to activate complement cascades, playing essential roles in lipid metabolism, inflammatory responses, and immune regulation (83). *Slc4a10*, predominantly expressed in the central nervous system, regulates bicarbonate transport and short-term synaptic plasticity, with aberrant expression potentially leading to GABAergic transmission deficits and associated neurological disorders (84, 85). We also identified *Homer3*, a synaptic plasticity regulator involved in G protein-coupled glutamate receptor signaling that associates with various malignancies including colon adenocarcinoma (86) and hepatocellular carcinoma (87). The gene exhibited a clear modulation trend by both EA and vagotomy across groups, though without reaching statistical significance. In contrast, *Vom2r44*, *RGD1566007*, and *Cd209e* remain poorly characterized in disease contexts, warranting further investigation.

Based on the WGCNA results and integrated multi-omics network analysis, we further focused on *Tnfrsf12a*, a TNF superfamily gene that regulates inflammation, tissue repair, and fibrosis through ligand Tnfsf12 binding. Among the identified EA- and VN-dependent DEGs, Tnfrsf12a emerged as a core hub gene within the MEfirebrick3 co-expression module, which exhibited significant activation in FD model rats, suppression following EA treatment, and reactivation after vagotomy, indicating a strong dependence on intact vagal signaling. Importantly, correlation analyses demonstrated robust positive associations between *Tnfrsf12a* expression and pro-inflammatory cytokines, including IL-6, IL-1β, and TNF-α, further supporting its involvement in duodenal low-grade inflammation. *Tnfrsf12a* activation modulates multiple signaling pathways including NF-κB, playing central roles in inflammatory responses. Current evidence indicates FD involves low-grade inflammation characterized by elevated TNF-α and IL-6 levels (45, 88, 89), consistent with our findings. We further investigated the TWEAK/Fn14/NF-κB signaling axis and found that EA treatment significantly reduced TWEAK, Fn14, and NF-κB p65 expression in MOD rats, an effect attenuated by vagotomy, demonstrating VN-dependent suppression of this pro-inflammatory pathway in FD. KEGG analysis of differential genes revealed significant enrichment in arachidonic acid metabolism, PPAR signaling, and complement and coagulation cascades.

We systematically integrated gut microbiota, transcriptomic, and metabolomic datasets to construct a microbiota–gene–metabolite regulatory network, enabling the identification of coordinated molecular alterations underlying EA intervention. Within this network, *Tnfrsf12a*-centered gene modules were tightly linked to arachidonic acid–related metabolites and inflammation-associated microbial taxa, indicating that EA ameliorated FD primarily through VN-mediated regulation of an interconnected immune–metabolic–microbial network, with arachidonic acid metabolism acting as a key downstream effector pathway. This ω-6 polyunsaturated fatty acid participates in immune-inflammatory responses, vascular function, and cellular signaling via COX, LOX, and cytochrome P450 metabolic pathways. While arachidonic acid dysregulation is well-established in diseases like IBD (90), liver cancer (91), and colorectal cancer (92, 93), its mechanistic role in FD remains to be fully elucidated. This study focused on the COX metabolic pathway’s role in FD pathogenesis. The pathway converts arachidonic acid to prostaglandins through two isoforms: constitutively expressed COX-1 (physiological PGE_2_ synthesis) and inducible COX-2 (pathological PGE_2_ production) (94). PGE_2_ exerts its biological effects through distinct receptor subtypes: EP1 mediates calcium-dependent pain perception and vasoconstriction while suppressing COX-2 expression; EP2/EP4 activate Gas protein and adenylate cyclase to initiate cAMP/PKA signaling; and EP3 reduces cAMP levels via Gi-coupled pathways, triggering diverse cellular responses (95). Anatomically, EP3 concentrates in the duodenal myenteric plexus, EP4 distributes to both epithelial surfaces and myenteric plexus, whereas EP2 shows limited villous presence and EP1 mRNA remains undetectable by *in situ* hybridization (96). Our results demonstrated that FD model rats exhibited significant upregulation of COX-2, PGE_2_ and EP4 compared to controls, while EP3 remained unchanged. EA effectively normalized these alterations, an effect abolished by vagotomy, confirming the VN’s pivotal role. In studies on the expansion and metastasis of colorectal cancer stem cells, PGE_2_ has been found to bind to its receptor EP4, thereby activating the downstream PI3K/AKT and MAPK/ERK signaling pathways, which in turn promotes the activation of NF-κB (97). Our study first established the arachidonic acid metabolic pathway’s involvement in FD, providing experimental support for EA’s clinical application.

This study has several limitations. First, the absence of sham acupuncture controls (non-acupoint shallow needling or mock EA)as well as the lack of other control groups—such as a sham model group (where 2% sucrose is administered during modeling), a sham surgery group (where vagotomy is simulated but not actually performed), and a sham surgery plus EA group (where vagotomy is simulated but not performed, while EA is still administered)—may affect the conclusiveness of the findings. The inclusion of these control groups would enable a more precise assessment of the specific effects of EA and vagotomy, helping to distinguish between true therapeutic effects and placebo responses. Second, while our focus was on EA’s peripheral effects via the VN and gastrointestinal function, we did not investigate central nervous system mechanisms (cortical activity or nuclei modulation), which limits a comprehensive understanding of EA’s systemic actions. Third, while our study identified two pathways suppressed by EA (TWEAK/Fn14/NF-κB and arachidonic acid metabolism), the potential crosstalk or hierarchical relationship between them remains unexplored. Furthermore, our study did not delve into the underlying neurobiological mechanisms of VN mediation, such as the specific subtypes of vagal neurons involved, the role of key neurotransmitters (e.g., acetylcholine), or the dynamic interplay within the broader brain-gut-immune-endocrine axis. Additionally, inherent differences between animal models and human disease necessitate validation through multicenter, large-sample randomized controlled trials. Future research should incorporate neuroimaging techniques such as fMRI to assess central regulation and conduct standardized clinical studies to facilitate translation.

## 5 Conclusion

This study identifies a novel dual-pathway mechanism underlying EA treatment for FD, centrally mediated by the VN. Through an integrated multi-omics approach, we demonstrate that EA simultaneously suppresses both the TWEAK/Fn14/NF-κB signaling pathway and arachidonic acid metabolism in a VN-dependent manner. This dual inhibition effectively enhances gastrointestinal motility, regulates duodenal immuno-inflammatory responses, and restores microbial-metabolic homeostasis. Our findings decipher a VN-coordinated therapeutic strategy for FD, establishing EA as a promising multi-target therapy with a clear mechanistic basis for clinical translation. However, several limitations—including the lack of specific control groups, the unexplored interaction between the dual pathways, and the unexamined neurobiological specifics of VN mediation—should be addressed in future studies. Further investigation integrating neurocircuit mapping and molecular crosstalk analysis will refine our understanding and optimize EA-based therapeutic strategies for FD.

## Data Availability

The data presented in the study are deposited in the NCBI Sequence Read Archive (SRA) and OMIX repositories. The 16S rDNA sequencing data are available under BioProject PRJNA1422414, the RNA-seq data under BioProject PRJNA1422499, and the metabolomics dataset under OMIX accession OMIX015135 (https://ngdc.cncb.ac.cn/omix). Detailed information is provided in **Supplementary Table 8**.

## Supplementary material

Supplementary Figure 1 Duodenal microbial analyses. **(A)** rarefaction curve, **(B)** rank-abundance, **(C)** ACE index, **(D)** Chao1 index, **(E)** Shannon index, **(F)** Simpson index, and **(G, H)** OPLS-DA analyses.

Supplementary Figure 2 The total ion chromatograms. **(A)** positive ionization mode, **(B)** negative ionization mode.

Supplementary Figure 3 KEGG pathway analysis of differential metabolites. **(A)** EA versus MOD groups, **(B)** SDV+EA versus EA groups.

Supplementary Figure 4 KEGG pathway analysis of differentially expressed genes. **(A)** EA versus MOD groups, **(B)** SDV+EA versus EA groups.
