# Supplementary material for: Electroacupuncture alleviates functional dyspepsia by modulating the vagus nerve to regulate duodenal microbiota and suppress TWEAK/Fn14/NF-κB and arachidonic acid metabolic pathways
Source: Front Immunol. 2026 Mar 9;17:1746351. doi: 10.3389/fimmu.2026.1746351 (PMC13010177; doi:10.3389/fimmu.2026.1746351)
Supplement: Supplementary Figure 1 — Duodenal microbial analyses. (A) rarefaction curve, (B) rank-abundance, (C) ACE index, (D) Chao1 index, (E) Shannon index, (F) Simpson index, and (G, H) OPLS-DA analyses. [file DataSheet1.docx]

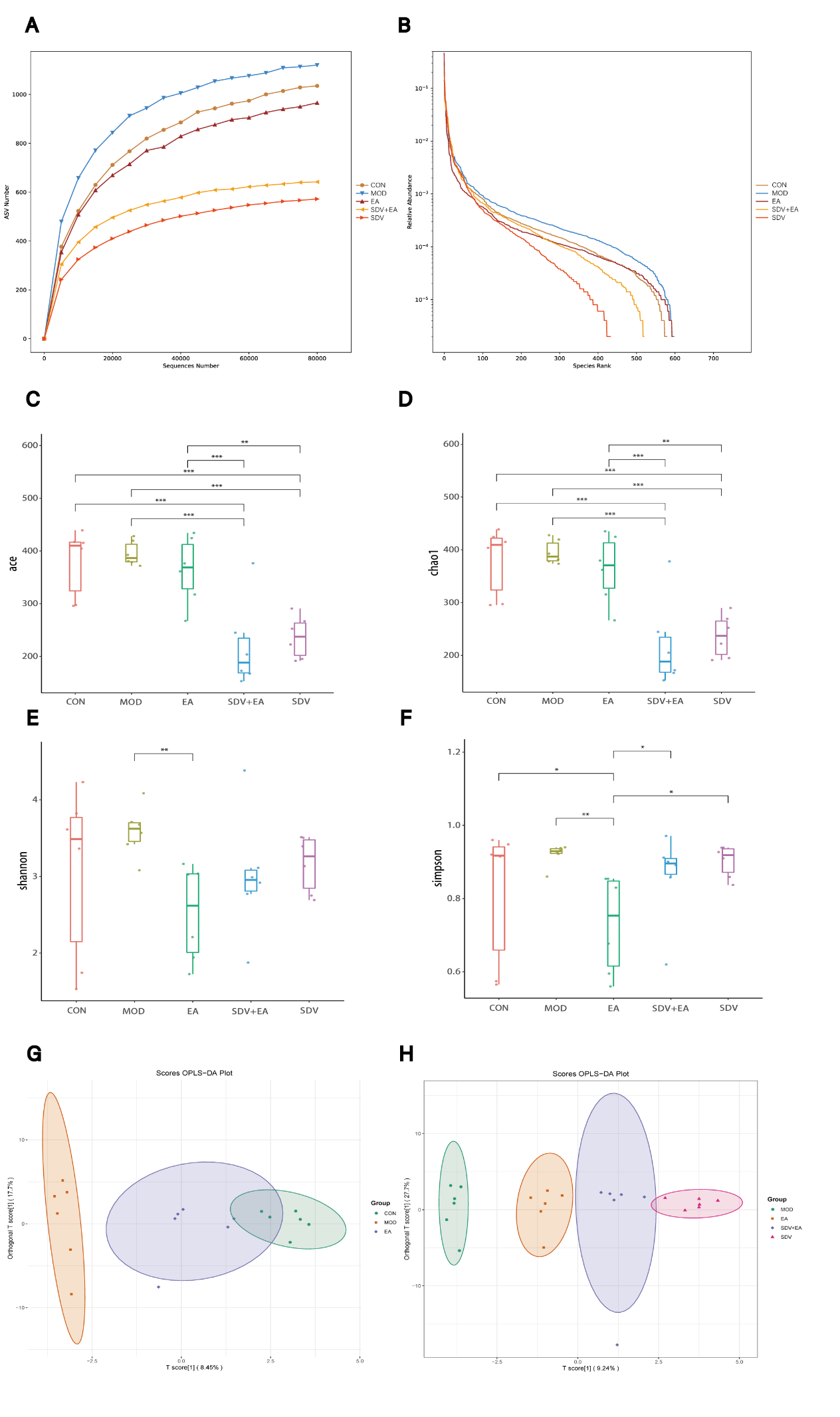


Figure 1. Duodenal microbial analyses. (A) rarefaction curve, (B) rank-abundance, (C) ACE index, (D) Chao1 index, (E) Shannon index, (F) Simpson index, and (G, H) OPLS-DA analyses.


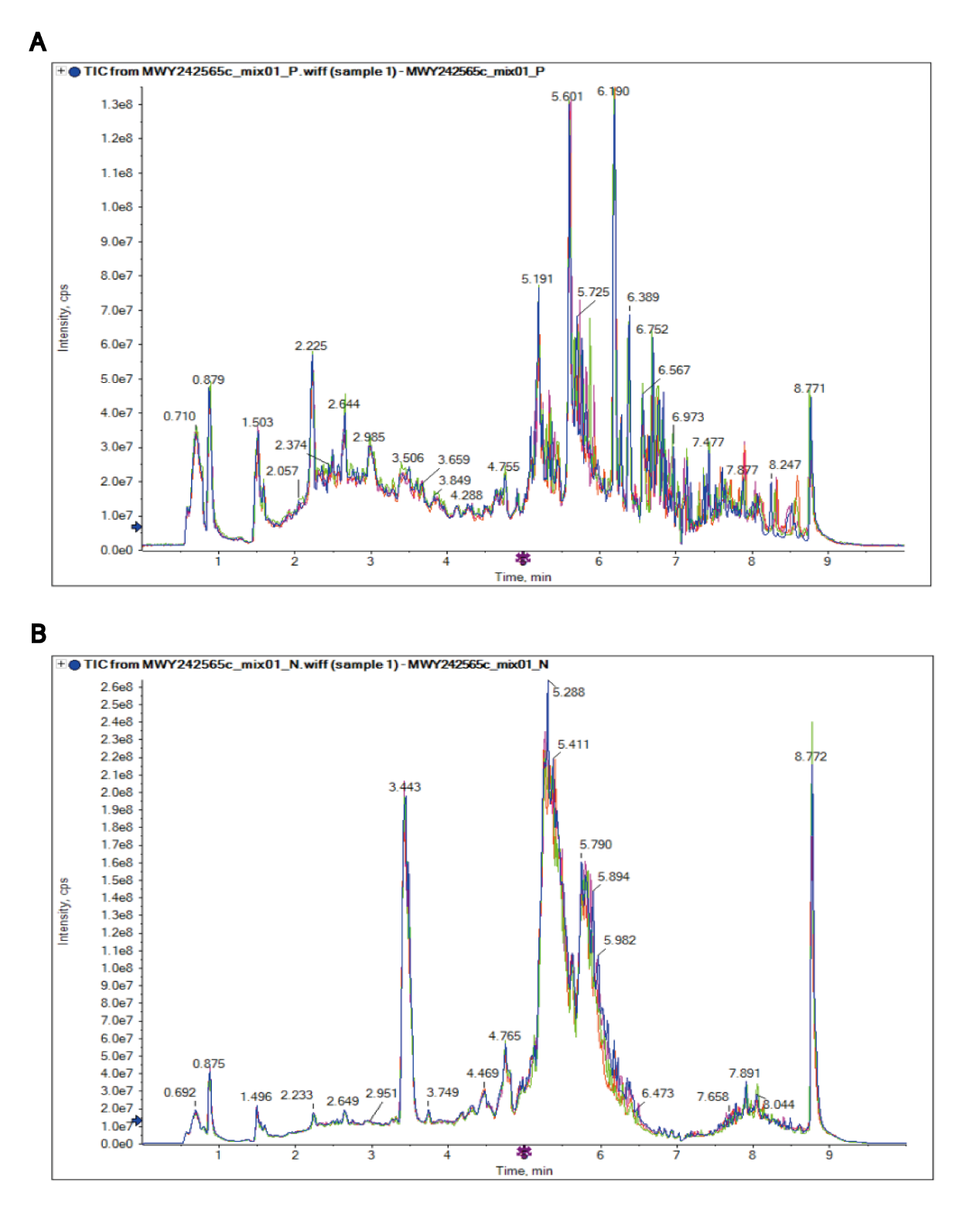


Figure 2. The total ion chromatograms. (A) positive ionization mode, (B) negative ionization mode.
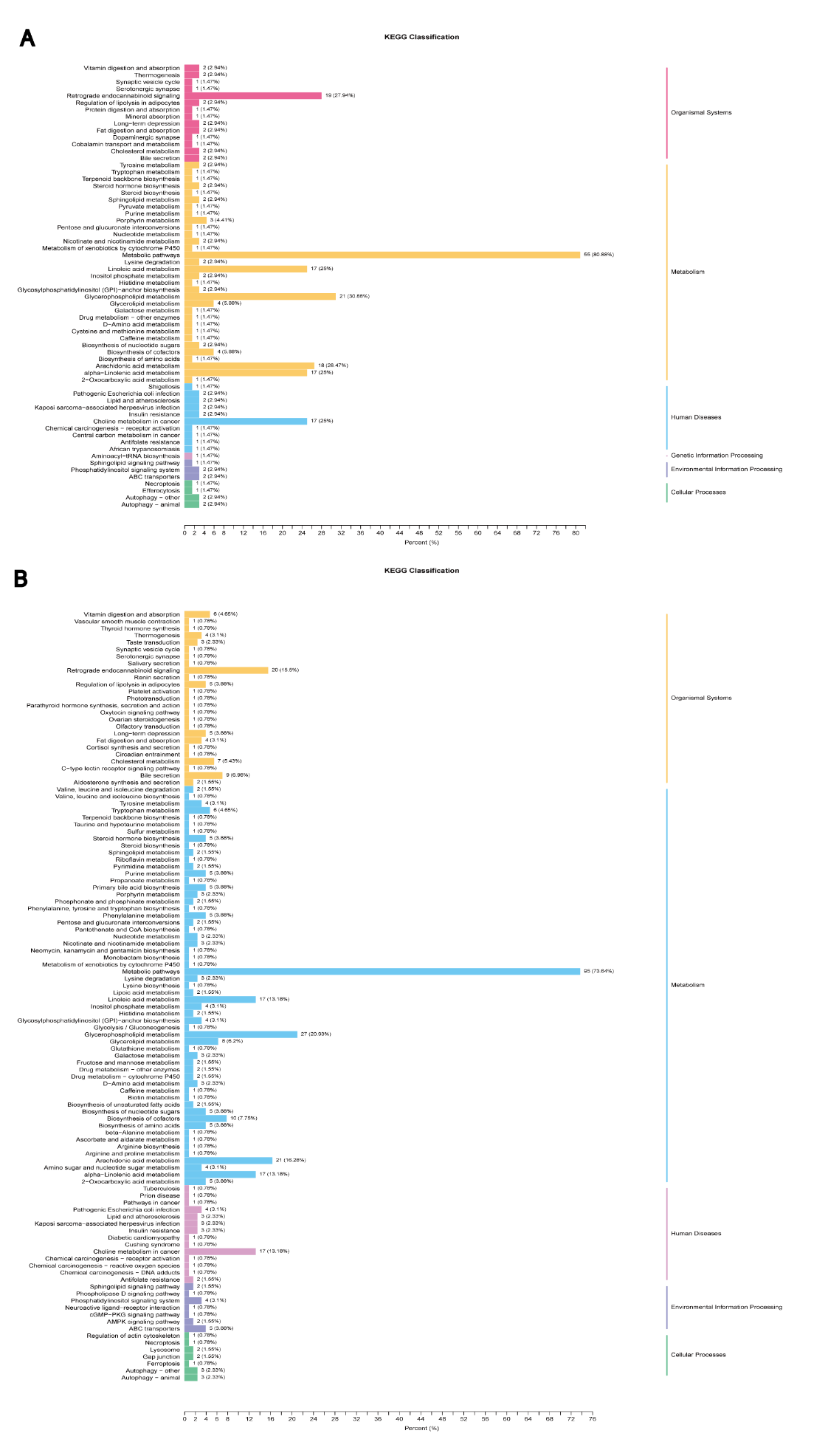


Figure 3. KEGG pathway analysis of differential metabolites. (A) EA versus MOD groups, (B) SDV+EA versus EA groups.


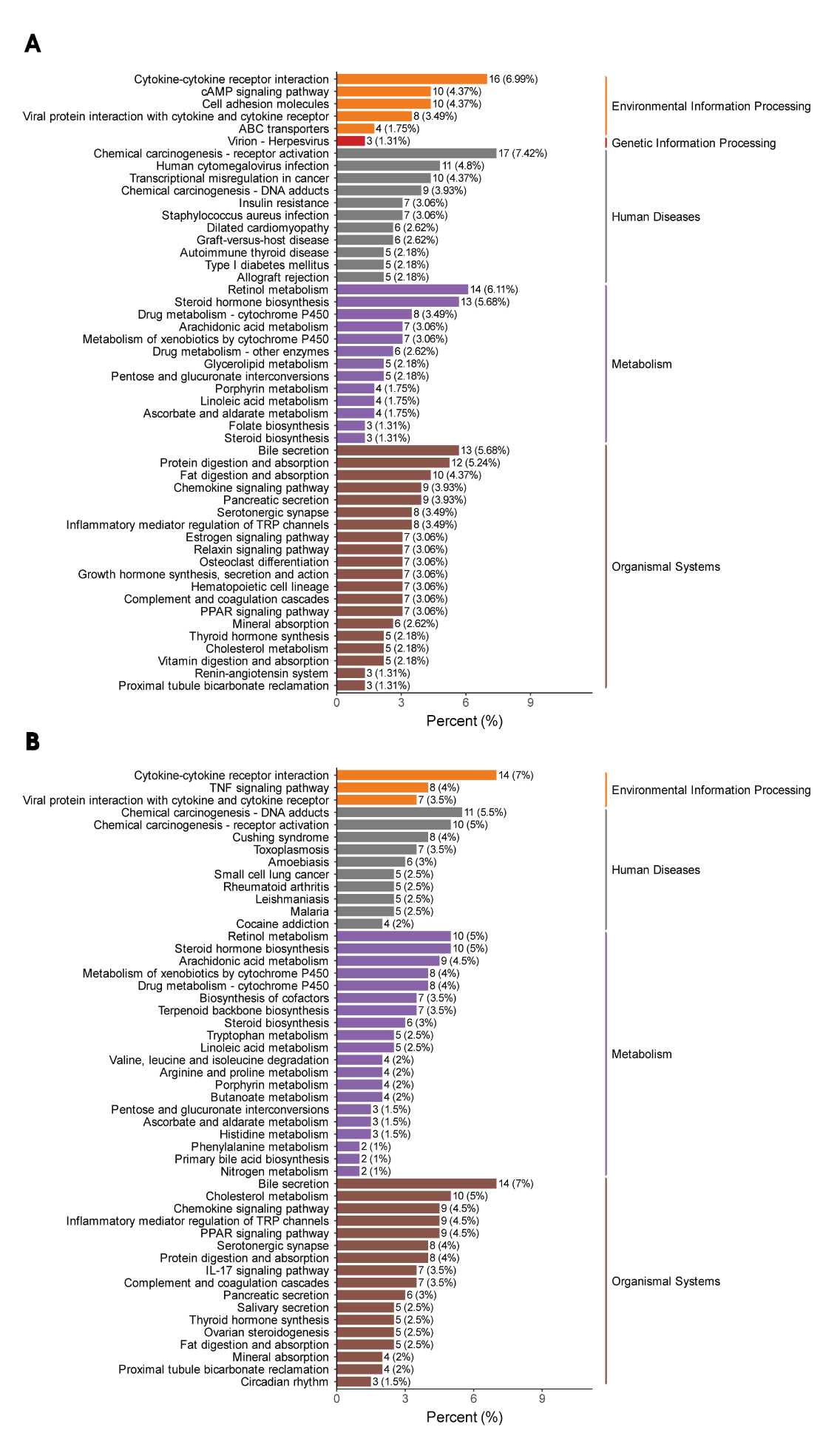


Figure 4. KEGG pathway analysis of differentially expressed genes. (A) EA versus MOD groups, (B) SDV+EA versus EA groups.
